# Dysfunctional glycolysis-UCP2-fatty acid oxidation promotes CTLA4^int^FOXP3^int^ regulatory T-cell production in rheumatoid arthritis

**DOI:** 10.1186/s10020-025-01372-6

**Published:** 2025-10-09

**Authors:** Jiawen Han, Zhongyang Zhou, Hongxia Wang, Yuxin Chen, Wuguo Li, Meiqin Dai, Jing Bian, Erming Zhao, Jiaying He, Xinyao Zhang, Huanfa Yi, Lan Shao

**Affiliations:** 1 The Center for Translational Medicine, The First Affiliated Hospital, Sun Yat-Sen University, Guangzhou, 510080 P. R. of China; 2 Laboratory Medicine Center, Nanfang Hospital, Southern Medical University, Guangzhou, 510515 P.R. of China; 3 Department of Rheumatology, Nanfang Hospital, Southern Medical University, Guangzhou, 510515 P. R. of China; 4 Department of Laboratory Medicine, Affiliated Hospital of Medical School, Nanjing Drum Tower Hospital, Nanjing University, Nanjing, 210008 P. R. of China; 5 Department of Urology, Henan Cancer Hospital, Zhengzhou, 450008 P.R. of China; 6 Central Laboratory, The First Hospital of Jilin University, Changchun, 130061 P.R. of China

**Keywords:** Fatty acid β-oxidation, Glycolysis, Caveolae, CTLA4, UCP2, Treg, Rheumatoid arthritis

## Abstract

In rheumatoid arthritis (RA), the impaired function of regulatory T cells (Tregs) induces chronic inflammatory responses in the synovium. Cytotoxic T Lymphocyte Antigen 4 (CTLA4) is crucial for Sustaining Treg function. Uncoupling Protein 2 (UCP2) plays a key role in linking the glycolysis and fatty acid oxidation (FAO) pathways. Here we observed that Tregs in RA were predominantly characterized by a CD25^int^CTLA4^int^FOXP3^int^CD4^high^ Phenotype. Mechanistically, elevated expression of UCP2 in RA Tregs disrupted metabolic homeostasis by downregulating Carnitine Palmitoyltransferase 2 (CPT2), a rate-limiting enzyme in the FAO pathway. Impaired FAO trigged caveolae-mediated endocytosis, leading to reduced CTLA4 cell surface accumulation and diminished Treg suppressive capacity. Moreover, UCP2 inhibition attenuated the pro-inflammatory effects of RA T cells in a human-SCID chimeric mouse model. Our results establish a critical link between CTLA4 endocytosis and UCP2-mediated metabolic shift in Tregs and identified UCP2 as a potential therapeutic target for preventing RA autoimmunity.

**Supplementary Information:**

The online version contains supplementary material available at 10.1186/s10020-025-01372-6.

## Introduction

Rheumatoid arthritis (RA) is an autoimmune disease characterized by chronic inflammation of the joint synovial membrane and infiltration of activated inflammatory cells (Harnden et al. 2016; Kondo et al. 2018). Regulatory T (Treg) cells play a critical role in mediating immunosuppression during the onset and progression of RA. Tregs express high levels of the Cytotoxic T Lymphocyte Antigen 4 (CTLA4), a key immune checkpoint molecule implicated in RA susceptibility as confirmed by multiple candidate gene-association studies (Davis et al. 2014). During RA, maldifferentiation of T cell lineages result in a deficient accumulation of CTLA4 on the cell Surface and insufficient expression of forkhead box protein 3 (FOXP3), leading to an increased production of CTLA4^int^FOXP3^int^CD4^high^ Tregs (Flores-Borja et al. 2008). Those findings underscore that the proper CTLA4 surface localization for Treg is indispensable for the suppressive function (Wing et al. 2008). The trafficking of CTLA4 from the cell surface to the cytoplasm is regulated by the endocytosis (Walker and Sansom 2011). However, the differences in the CTLA4 endocytosis patterns between patients with RA and healthy controls (HCs) are remain inadequately defined.

Upon activation, T cells undergo proliferation and differentiation, necessitating metabolic reprogramming to meet their elevated bioenergetic and biosynthetic demands. This process involves various metabolic pathways. Fatty acid β-oxidation (FAO) is the primary process associated with the degradation of fatty acids to generate energy. To enter the mitochondria for oxidation, fatty acids are first conjugated to free carnitine by carnitine palmitoyltransferase I (CPT1). This complex is then shuttled through the mitochondrial membrane, where CPT2 catalyzes the release of fatty acid acyl-CoA groups from carnitine (Schlaepfer and Joshi 2020; Wong et al. 2017). Therefore, CPT1 and CPT2 are the rate-limiting enzymes in the FAO pathway. Compared to effector T cells, Tregs exhibits increased levels of fatty acid transporters. This metabolic adaptation favors the FAO metabolism over glycolysis to fulfill their energy requirements (Maciolek et al. 2014; Raud et al. 2018). In RA, CD4^+^ T cells display significant metabolic dysregulation (Weyand and Goronzy 2021, 2017), including impaired glucose utilization and excessive fatty acid accumulation (Shen et al. 2017). Although cellular energy-sensing system typically balances glycolysis and fatty acid metabolism, RA T cells exhibit concurrent defects in both pathways, suggesting a systemic failure in metabolic regulation.

Mitochondrial uncoupling proteins (UCPs) are integral components of the inner mitochondrial membrane that dissipate the proton gradient, reducing the membrane potential and releasing stored energy as heat. The major components of the UCP family include UCP1 and its homologs UCP2 and UCP3 (Krauss et al. 2005). Among them, *UCP2* has been identified as a susceptible gene for RA and is abundantly expressed in activated proliferating T cells (Chaudhuri et al. 2016). UCP2 mediates proton leakage and controls the production of superoxide radicals and other downstream reactive oxygen species (Yoon et al. 2022; Kim et al. 2019; Zhang et al. 2020). Beyond its classical uncoupling functions, emerging evidence indicates that UCP2 activation orchestrates a metabolic shift from mitochondrial oxidative phosphorylation to glycolysis while promoting lipid synthesis (Xu et al. 2017). These findings suggests that UCP2 sustains glycolytic flux by suppressing lipid metabolism.

In this study, we observed that the Treg population being predominantly CD25^int^CTLA4^int^FOXP3^int^CD4^high^ Tregs rather than the canonical CD25^high^CTLA4^high^FOXP3^high^CD4^high^ Tregs in RA. Mechanistically, RA-derived CD4^+^ T cells exhibited elevated UCP2 expression, which consistently suppressed FAO metabolism by downregulating CPT2. Consequently, lipid rafts accumulated in the cell surface membrane, coupled with the activation of caveolae-mediated CTLA4 endocytosis. As a result, Treg immunosuppressive functions were impaired in RA. Our findings demonstrated that the increased production of CD25^int^CTLA4^int^FOXP3^int^CD4^high^ Tregs was associated with an atypical UCP2-linked metabolic switch from FAO to glycolysis, identifying UCP2 as a potential therapeutic target for mitigating autoimmunity in RA.

## Material and methods

### Patients and control individuals

The study group included 71 individuals diagnosed with RA and 72 healthy donors (HCs). The patients with RA fulfilled the ACR/European League Against Rheumatism (EULAR) 2010 classification criteria for RA (Aletaha et al. 2010), and all patients with RA were positive for rheumatoid factor and/or anticyclic citrullinated peptide antibody. The control subjects were matched with age, gender and ethnicity. A history of cancer, uncontrolled medical disease or any other inflammatory syndrome were excluded. Healthy individuals did not have any personal or family history of autoimmune disease. The demographic characteristics of the patients with RA and HC donors are summarized in Table 1. The study was approved by the Medical Ethics Committee of Hospital and all subjects provided appropriate informed consent.

**Table 1:** Demographic and clinical characteristics of the study populations

Characteristics	HC	OA	RA	*P*
Demographic				
Number of subjects	72	12	71	
Female/Male^a^	56/16	9/3	54/17	0.74
Age (mean ± SD years)^a^	51.7 ± 2.8	52.9 ± 1.7	53.1 ± 2.1	0.31
Disease status				
Disease duration (mean ± SD years)		N/A	9.2 ± 2.0	
Active disease		N/A	87.3%	
Tobacco use		N/A	7.0%	
Extra-articular manifestations		N/A	46.5%	
ESR, mm/h		N/A	37.2 ± 3.7	
DAS28-CRP (mean ± SD)		N/A	3.7 ± 0.7	
DMARD naïve		N/A	9.9%	
Medications				
Corticosteroids		N/A	73.2%	
Methotrexate		N/A	67.6%	
Hydroxychloroquine		N/A	54.9%	
Leflunomide		N/A	23.9%	
TNF-α inhibitors		N/A	12.6%	

*ESR* Erythrocyte sedimentation rate, *DAS28* Disease Activity Score in 28 joints, *DMARD* Disease-modifying antirheumatic drugs, an active disease defined by Food and Drug Administration (FDA) criteria [presence of three or more of the following: morning stiffness (> 45 min), swollen joints (> 3), tender joints (> 6) and sedimentation rate (> 20 mm/h)]

^a^No significant difference, RA patients compared with HC donors

### Cell purification and culture

To sort CD4^+^CD45RO^−^ cells, Peripheral blood mononuclear cells (PBMCs) were negatively selected with CD45RO microbeads (130–046-001, Miltenyi Biotec Inc., Auburn, USA), followed by positive selection with CD4 micro-beads (130–097-048, Miltenyi Biotec Inc.) using autoMACS (130–097-048, Miltenyi Biotec Inc.). CD4^+^CD45RO^−^ T cells (1.0** × **10^5^/well) were stimulated with anti-CD3/CD28-coupled beads (11132D, Invitrogen, Carlsbad, USA) in a 2.5:1 ratio, supplemented with IL-2 (20 IU/ml, # 200–02, Peprotech, Rocky Hill, USA).

For Treg differentiation, CD4^+^CD45RO^−^ T cells were stimulated with anti-CD3/CD28-coupled beads, supplemented with IL-2 (10 ng/ml, # 200–02, Peprotech) and TGF-β1 (10 ng/ml, # 100–21, Peprotech), for 5 days (Su et al. 2019).

For suppression assays, CD4^+^CD25^+^ Tregs and CD4^+^CD25^−^ T responder cells were isolated using a Treg isolation kit (130–093-631, Miltenyi Biotec Inc.) and co-cultured at a 1:4 ratio in 96-well plates. Cells were then stimulated with anti-CD3/CD28-coupled beads, and after 4 days, stained with CD4-APC (#300,514, Biolegend, San Diego, USA) and PE-CD25 (#302,610, Biolegend) antibodies to distinguish each T-cell population. Proliferation of CD4^+^CD25^−^ T cells was quantified by CFSE Cell Division Tracker Kit (#423,801, Biolegend).

For the inhibitor experiments, cells were treated with the following compounds for 24 h on day 3 after Treg differentiation: 10 mM Methyl-β-cyclodextrin (#128,446–36-6, TargetMol, Shanghai, China), a caveolar inhibitor; 10 μM Pitstop2 (#1,419,320–73-2, TargetMol), a clathrin inhibitor; 20 μM Etomoxir (HY-50202, MedChemExpress, Monmouth Junction, USA), a CPT1A-specific inhibitor; and 25 μM Genipie (HY-17389, MedChemExpress), a UCP2-specific inhibitor.

### In vitro Treg suppress B-cell differentiation

CD4^+^CD45RO^−^ T cells were cultured under Treg-polarizing conditions. On day 5, CTLA4^high^CD127^low^ and CTLA4^low^CD127^low^ Tregs were sorted using an LSRFORTESSA X-20 flow cytometer (Becton, Dickinson & Company, San Jose, USA), and then co-cultured with B cells isolated from an unrelated donor, and stimulated with SEB (1 μg/mL; s4881, Sigma-Aldrich, St. Louis, USA) for 7 days in 96-well plates, either uncoated or pre-coated with Recombinant anti-Human CD3 (GMP-A018, Novoprotein, Su Zhou, China) and anti-Human CD28 (GMP-A063, Novoprotein) antibodies.

### Flow cytometry

For flow cytometry analysis, cells were harvested and washed twice with FACS buffer. The following fluorochrome-conjugated antibodies from BioLegend were used for staining: CD4-APC (Clone RPA-T4, #300,514), CD4-PE-Cy7 (Clone SK3, #344,611), CD45RA-PE (Clone HI100, #304,108), CD45RA-FITC (Clone HI100, #304,106), CD25-APC (Clone BC96, #302,610), CD25-PE (Clone BC96, #302,606), CTLA4-APC (Clone BNI3, #369,612), CTLA4-PE (Clone L3D10, #349,905), CTLA4-PerCP-Cy5.5 (Clone BNI3, #369,608), CD40L-PE (Clone 24–31, #310,805), ICOS-FITC (Clone C398.4A, #313,505), CD19-APC (Clone HIB19, #302,211), CD19-PE (Clone HIB19, #302,207), CD38-FITC (Clone HIT2, #303,503), IgD-PE-Cy7 (Clone IA6-2, #348,209), and FOXP3-FITC (Clone 206D, #320,105). Primary antibodies against UCP2 (sc-390189, Santa Cruz Biotechnology), CPTI (sc-393070), Clathrin Heavy Chain/CLTC (sc-12734), Caveolin-1(sc-70516), PFKFB3 (sc-293477), IL-6 (#701,028, eBioscience, USA), TNF-α (#MA5-23,720, eBioscience), IL-10 (#AHC0102, eBioscience), and TGF-β (#MA1-21,595, eBioscience) were used. Flow cytometry analysis was performed using CytoFLEX flow cytometer (Becton, Dickinson & Company). Data was analyzed using FlowJo 10.4.

### Enzyme-Linked Immunosorbent assay (ELISA)

Cytokine levels were quantified using ELISA kits specific for human IL-10 (#H980288, Macklin, China), IFN-γ (#H980359, Macklin), and TNF-α (#H980361, Macklin) in accordance with the manufacturer’s instructions. Cell culture Supernatants were incubated in 96-well plates pre-coated with antibodies against IL-10, IFN-γ, and TNF-α. Detection was performed using biotinylated antibodies specific for each cytokine, followed by incubation with horseradish peroxidase (HRP)-conjugated streptavidin. Absorbance was measured at 450 nm using a Thermo Scientific ™ Varioskan ™ LUX multimode reader (N16045, Thermo). Sample concentrations were determined based on the curves generated from known standards.

### Immunofluorescence and immunohistochemistry

Treg cell were fixed in 4% paraformaldehyde for 15 min, permeabilized using 0.2% triton X-100 for 10 min followed by staining with rabbit anti-human CTLA4, anti-human Clathrin Heavy Chain/CLTC (sc-12734, Santa Cruz Biotechnology), and mouse anti-human Caveolin-1 (sc-70516) or normal control IgG 6–8 h at 4 °C. Then, Alexa Flour 488-conjugated goat anti-rabbit IgG and Alexa Flour 594-conjugated goat anti-mouse IgG were added and incubated for 1 h at 37 °C. The nucleus was stained with DAPI and mounted with ProLong Gold Antifade Reagent. The fluorescence intensity was quantified via the LSM 880 colocalization function (Zeiss, Jena, Germany).

### Quantitative real-time PCR (qPCR)

Total RNA was extracted from 1 × 10^5^ cells, and cDNA was synthesized using AMV-reverse transcriptase and random hexamer primers (Roche Diagnostic Corp., Indianapolis, USA). Reverse transcription was performed using a standard procedure (Super Script First-Strand Synthesis System; Invitrogen). qPCR was performed on a StepOne Plus Real-Time Detection system (Applied Biosystems, USA) with primer sequences were listed in Supplementary Table 1. Relative amount of mRNA was calculated using the 2^−ΔCt^. 18S ribosomal RNA was used as housekeeping gene.

### Immunoprecipitation and western blotting

CD4^+^ T cells were washed twice with cold PBS and lysed in 1.5 ml of cold lysis buffer. The supernatants of the cell lysates were precleared by incubation with protein A/G PLUS-Agarose for 2 h. Then, the lysates were incubated with anti-CTLA4 (sc-18829, Santa Cruz Biotechnology) or CPT2 (sc-377294) and protein A/G PLUS-Agarose on a rotator at 4 °C for 12 h. Immune complexes were collected after each immunoprecipitation by centrifugation at 13,000 × g for 10 min. The immune complexes were subjected to sodium dodecyl sulfate–polyacrylamide gel electrophoresis (SDS-PAGE), followed by immunoblotting with Clathrin Heavy Chain/CLTC (sc-12734), Caveolin-1 (sc-70516) and CTLA4 (sc-18829), and anti-Acetylated-Lysine Ac-K-103 (#9441, Cell signaling Technology, Danvers, USA) antibodies.

T cells were lysed in an ice-cold RIPA buffer (Beyotime, P0013B, Shanghai, China) (Shao et al. 2009). Equal amounts of total protein from each sample were separated by SDS-PAGE gel and then transferred to a nitrocellulose membrane. Membranes were probed overnight at 4 °C with primary antibodies against CTLA4 (sc-18829), UCP2 (sc-390189), CPT1A (DF12004, Affinity Biosciences) and CPT2 (sc-377294), followed by incubation with secondary antibodies for 1 h at room temperature. Protein bands were visualized using a chemiluminescent detection system (Beyotime, P0018S).

### Fatty acid oxidation assay

Oleate was used as substrate to evaluate fatty acid-driven oxygen consumption in CD4^+^ T cells according to the manufacturer’s instructions (ab222944, Abcam, Cambridge, USA). CD4^+^CD45RO^−^ T cells (1.0** × **10^5^/well) were seeded in a Costar 96 well plate and were stimulated with anti-CD3/CD28-coupled beads. Cells treated with FCCP served as the positive control, while etomoxir for the negative controls. The oxygen consumption rate was measured using a BioTek Synergy H1 Multimode Reader (Agilent, USA) and was expressed as the initial rate of increase in the fluorescent intensity.

### UCP2 knockdown and overexpression

CD4^+^CD45RO^−^ T cells were stimulated with anti-CD3/CD28-coupled beads for 3 days, and then UCP2 siRNA and control siRNA, or mCherry-PRP and mCherry-UCP2 plasmids (VectorBuilder, Guangzhou, China) were transfected into the cells using NEPA21 electroporator (Nepa Gene Co., Ltd., Japan), according to the manufacturer’s protocol.

### Synovitis induction in chimeric mice

Female NOD.Cg-PrkdcscidIl2rgtm1Wjl/SzJ (NSG) mice (10–14 weeks old; The Jackson Laboratory, USA) were used (Seyler et al. 2005). Pieces of synovial tissue from RA patients were implanted into a Subcutaneous pocket on the upper dorsal midline. For the inhibitor experiment, the mice were intravenously injected with 10 million CD45RO^−^ PBMCs isolated from RA patients. Littermate chimeric mice carrying the same synovial tissue were randomized into two groups: either treated with vehicle or with Genipin (4 mg/kg/day). Both treatments were administered via daily intraperitoneal injection for 9 days. For the UCP2 overexpression experiment, CD45RO^−^ PBMCs from HCs were transfected with either control or a UCP2 plasmid. At the end of the experiments, the mice were sacrificed, and the synovial tissues were harvested for paraffin embedding and histological analysis (Li et al. 2016).

### Synovial histopathological examination and immunostaining

Sections were either stained with haematoxylin and eosin (H&E) for histological evaluation or processed for immunofluorescence staining. For immunofluorescence, tissue sections were incubated with primary antibodies against CD3 (#ab21703, Abcam), CTLA4 (sc-18829), Caveolin-1 (sc-70516), and CPT2 (sc-377294) followed by fluoresce-conjugated secondary antibodies. Positive-staining cells were determined by manual counting observing 9 different fields of synovial tissue sections at magnification of × 400.

### Statistical analysis

Statistical analyses were performed using Prism software (version 9.0, GraphPad Software). Groups comparisons were conducted using parametric t-tests for independent or paired samples, as appropriate. Categorical variables between different groups were compared using the chi-square test. All data are presented as mean ± SEM. p < 0.05 was regarded as significant.

## Results

### Increase in CTLA4^int^FOXP3^int^ Treg production in patients with RA

**Fig. 1:**
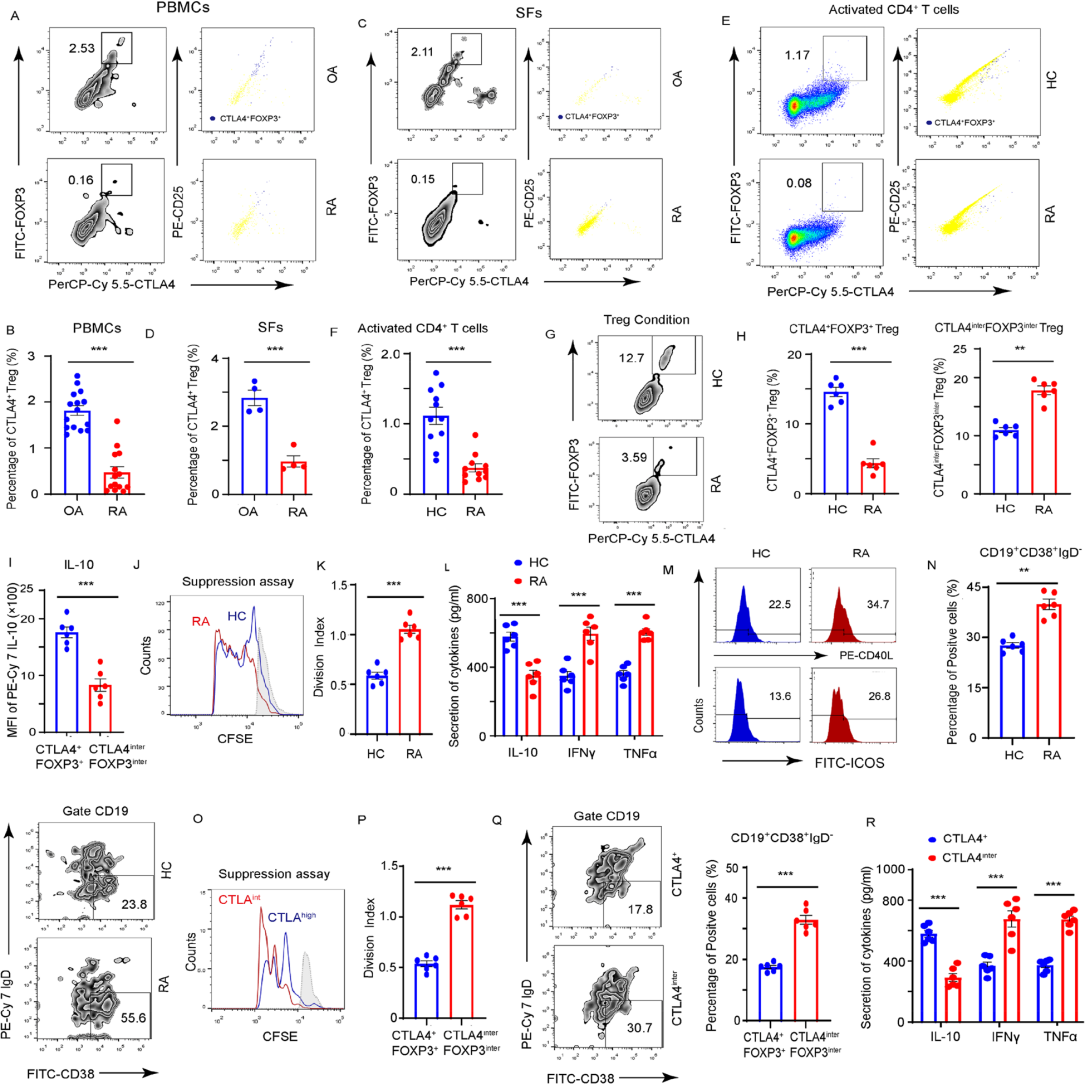
Increased CTLA4^int^FOXP3^int^ Treg production in patients with RA. **A**-**D** CTLA4^high^ Treg subsets among PBMCs and SFs from patients with RA and OA. **A** Representative plots for PBMCs. **B** Percentages of CD25^high^CTLA4^high^FOXP3^high^CD4^high^ Tregs among PBMCs examining 15 RA and 15 OA patients. **C** Representative plots for SFs. **D** Percentages of CD25^high^CTLA4^high^FOXP3^high^ CD4^high^ Tregs among SFs examining 4 RA and 4 OA patients. **E–F** CD4^+^ T cells from RA patients and healthy controls (HCs) were stimulated with anti-CD3/CD28 beads for 5 days, and **E** Representative plots. **F** Percentages of CD25^high^CTLA4^high^FOXP3^high^CD4^high^ T cells examining 11 RA patients and 11 HCs were presented. **G**–**R** T cell differentiation: CD4^+^CD45RO^−^ T cells were cultured under Treg-polarizing conditions. **G**-**H** Percentages of CD25^high^CTLA4^high^FOXP3^high^CD4^high^ and CD25^inter^CTLA4^inter^FOXP3^inter^CD4^high^ Tregs under Treg-polarizing conditions examining 6 RA patients and 6 HCs were presented. **I** Expression of IL-10 was measured by FACS. **J**-**L** Tregs isolated from RA patients and HCs co-cultured with CFSE-labeled CD4^+^CD25^−^ T cells and stimulated with anti-CD3/CD28 beads for 5 days. **J** Proliferation of the T cells was analyzed by quantifying CFSE dilution at (**K**). **L** Cytokine Secretion was measured by ELISA. **M** Tregs isolated from RA patients and HCs cocultured with conventional CD4^+^ T cells. CD40L and ICOS levels in CD4^+^ T cells were analyzed by flow cytometry. **N** B cells isolated from same donors were divided into two parts and cocultured with either RA- or HCs-derived Tregs. Cultures were examined for the frequencies of CD19^+^CD38^high^IgD^low^ B cells. **O**-**R** CD25^high^CTLA4^high^FOXP3^high^CD4^high^ and CD25^inter^CTLA4^inter^FOXP3^inter^CD4^high^ Tregs were sorted from same RA patients and cocultured with CFSE-labeled CD4^+^CD25^−^ T cells. **O**-**P** Proliferation of the CD4^+^ T cells were analyzed by quantifying CFSE dilution. **Q** CD25^high^CTLA4^high^FOXP3^high^CD4^high^ and CD25^inter^CTLA4^inter^FOXP3^inter^CD4^high^ Tregs were cocultured with B cells. Frequencies of CD19^+^CD38^high^IgD.^low^ B cells were examined. **R** The production levels of cytokines were measured by ELISA. All data were presented as the mean ± SEM. Paired Student t-test, **p < 0.01; ***p < 0.001

To elucidate the phenotypes of Tregs during autoimmune inflammation, the frequency of CD25^high^CTLA4^high^FOXP3^high^CD4^high^ Tregs in PBMCs and synovial fluids (SFs) of patients with RA were examined using flow cytometry. Patients with Osteoarthritis (OA) were included as controls to exclude non-immune regulatory factors affecting Treg differentiation. The proportion of CD25^high^CTLA4^high^FOXP3^high^CD4^high^ Tregs was substantially reduced in both PBMCs (Fig. 1A and B) and SFs (Fig. 1C and D) of patients with RA compared to OA controls. Notably, CTLA4 surface expression strongly correlated with FOXP3 levels, underscoring the importance of CTLA4 localization for Treg differentiation and function.

Furthermore, upon anti-CD3/CD28 stimulation, the frequency of CD25^high^CTLA4^high^ FOXP3^high^CD4^high^ Tregs among CD4^+^ T cells was lower in RA than that in HCs (Fig. 1E and F), indicating impaired Treg differentiation in RA. To further explore whether the Tregs maldifferentiation was associated with RA, CD4^+^CD45RO^−^ naive T cells from patients with RA and matched HCs were isolated and induced differentiation under Treg-polarizing conditions. RA-derived Tregs exhibited a reduced population of CD25^high^CTLA4^high^FOXP3^high^ CD4^high^ subset combined with an increased population of CD25^int^CTLA4^int^FOXP3^int^CD4^high^ subset with lower IL-10 expression compared to HCs (Fig. 1G-I), indicating disrupted CTLA4 surface accumulation in RA Tregs.

Functional assays revealed that RA-derived Tregs exhibited diminished suppressive capacity compared to HCs. Co-culture of CFSE-labeled CD4^+^ T cells with Tregs showed that Tregs derived from RA were less effective at inhibiting T cell proliferation and effector cytokine secretion (Fig. 1J-L).

Similarly, RA Tregs displayed reduced suppression of B cell activation, as evidenced by significantly increased surface expression of CD40L and inducible T–cell costimulatory (ICOS) of CD4^+^ T cells (Fig. 1M), a higher frequency of CD19^+^CD38^high^IgD^low^ B cells (Fig. 1N), and elevated autoantibody production (Supplementary Table S2) than those of HCs. Moreover, CD25^high^CTLA4^high^FOXP3^high^CD4^high^ Tregs exhibited much stronger suppressive activity compared to CD25^int^CTLA4^int^FOXP3^int^CD4^high^ Tregs isolated from the same patients with RA (Fig. 1O–R), further supporting critical role of CTLA4 surface expression in Treg-mediated immunosuppression.

### Increase in caveolar expression promotes CTLA4 endocytosis in RA T cells

**Fig. 2:**
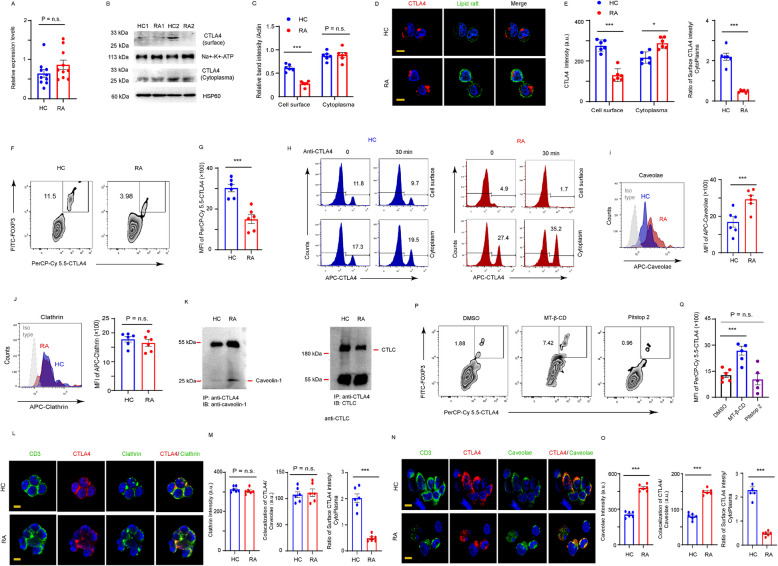
An increase in caveolar expression promotes CTLA4 endocytosis in RA Tregs. CD4^+^CD45RO^−^ T cells from RA patients and HCs were stimulated with anti-CD3/CD28 beads. **A** Expression of *CTLA4* in the T cells from RA patients and HCs on day 2 after CD3/CD28 stimulation were measured by qPCR. **B** Surface and cytoplasm distribution of CTLA4 was detected by western blotting. A representative blot from three experiments is shown and quantified at (**C**). **D**-**E** Cell surface distribution of CTLA4 was detected by CTLA4 staining (red) and T cell membrane was shown by lipid raft staining by cholera toxin Subunit B. Bar, 20 μm. **D** Representative images from 3 experiments were qualified at (**E**). **F**-**Q** T cell differentiation: CD4^+^CD45RO^−^ T cells were cultured under Treg-polarizing condition. **F**-**G** The CTLA4 Surface expression levels in Treg were analyzed by FACS on day 5. **H** CTLA4 endocytosis. CTLA4 Surface and cytoplasm distribution at 30 min after CTLA4 antibody stimulation were detected by FACS, representative plots were from 6 RA and 6 HCs. **I** Intracellular caveolar levels were detected by FACS and data from 6 RA patients and 5 HCs were quantified. **J** Intracellular clathrin level were detected by FACS. **K** Proteins from RA and HCs Tregs were subjected to immunoprecipitation with CTLA4 antibody followed by blotting with and caveolin-1 or clathrin antibodies. **L**-**O** CTLA4 (red) endocytosis patterns were determined with co-staining with caveolin-1 (green) and clathrin (green) antibodies. Fluorescence intensities of colocalization signal of CTLA4-clathrin and CTLA4-caveolae examining 6 HCs and 6 RA were presented. Bar, 20 μm. **P**-**Q** CD4^+^CD45RO^−^ T cells cultured under Treg-polarizing condition were treated with caveolar inhibitor Methyl-β-cyclodextrin (MT-β-CD, 10 mM) or clathrin inhibitor Pitstop2 (10 µM) for 24 h on day 3. The CTLA4 surface expression levels of Tregs were analyzed by FACS. All data were presented as the mean ± SEM. *p < 0.05; ***p < 0.001; n.s., non-significance

To assess CTLA4 surface expression in naive T cells, CD4^+^CD45RO^−^ T cells were isolated from patients with RA and matched HCs, followed by activation with anti-CD3/CD28 beads. qPCR analysis revealed comparable *CTLA4* expression in RA and HC-derived CD4^+^ T cells (Fig. 2A). However, a decreased in CTLA4 on the cell membrane was observed in CD4^+^ T cells isolated from RA via western blotting (Fig. 2B and C). Immunofluorescence co-staining of CTLA4 and lipid rafts (labeled with cholera toxin subunit B) further confirmed diminished CTLA4 surface retention in RA T cells (Fig. 2D and E).

To investigate whether this defect extended to Tregs in RA, CD4^+^ T cells from patients with RA and HCs were polarized into Tregs. CTLA4 surface expression was significantly lower in RA Tregs compared to that in HCs (Fig. 2F and G). To further determine whether the production of CTLA4^int^ Tregs in RA was associated with differences in endocytosis patterns, CTLA4 trafficking patterns in RA and HC Tregs were compared using fluorescence-activated cell sorting (FACS). Following CTLA4 antibody stimulation, CTLA4 internalization from the cell membrane to the cytoplasm occurred more rapidly in RA Tregs than that in HCs (Fig. 2H).

To identify the dominant endocytic pathway in Tregs, the expression of caveolae and clathrin in Tregs derived from RA patients and HCs were compared. Flow cytometry analysis revealed no significant difference in clathrin levels between RA and HC Tregs on day 5 post-polarization. In contrast, RA Tregs produced substantially more caveolae than HCs (Fig. 2I and J). The co-immunoprecipitation (co-IP) assay demonstrated a significant increase in caveolae co-precipitation with CTLA4 antibody in RA samples compared to HCs (Fig. 2K). Immunofluorescence staining further confirmed enhanced caveolar expression and colocalization of CTLA4 within caveolar endocytic vesicles (Fig. 2L–O). To investigate whether CTLA4 endocytosis in RA is predominantly mediated by caveolae, RA-derived CD4^+^ T cells were treated with inhibitors targeting caveolae- and clathrin-mediated endocytosis pathways. Notably, inhibition of caveolae-mediated endocytosis resulted in a significant increase in both CTLA4 surface localization and Treg differentiation (Fig. 2P and Q), suggesting that CTLA4 internalization in RA Tregs primarily occurred through caveolae-dependent mechanism.

### Inadequate FAO pathway induces caveolae-mediated CTLA4 endocytosis

**Fig. 3:**
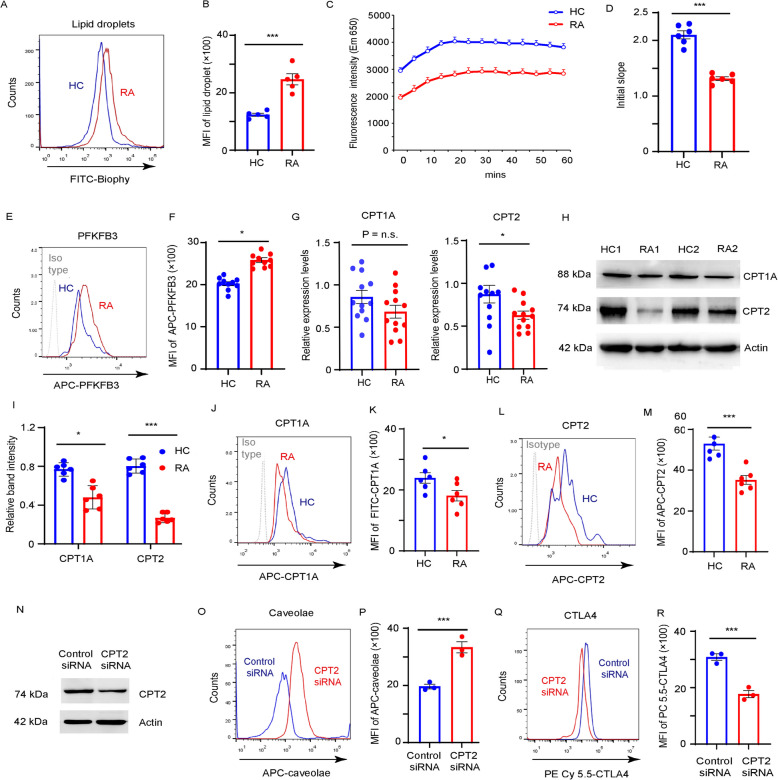
Inadequate FAO pathway induces caveolae-mediated CTLA4 endocytosis**.** CD4^+^CD45RO^−^ T cells from RA patients and HCs were stimulated with anti-CD3/CD28 beads and cultured under Treg-polarizing condition. **A**-**B** Neutral Lipid droplets were stained with Nile red and data examining 5 RA patients and 5 HCs were quantified at (**B**). **C**-**D** Time course and rate of increase in oleate-driven Fatty acid β-oxidation (FAO) assay. The data was recorded every 5 min. T cells treated with FCCP were used as positive control, and cells treated with Etomoxir were used as negative control. Relative FAO rate analyzing 6 RA patients and 6 HCs were quantified at (**D**). **E**–**F** PFKPB3 expression was detected by FACS and results analyzing 10 RA patients and 10 HCs were quantified. **G** *CPT1A* and *CPT2* gene expression in Tregs from RA patients and HCs. **H**-**I** Representative blotting for CPT1A and CPT2 protein expression. Results analyzing 6 RA patients and 6 HCs were quantified at (**I**). **J**-**M** CPT1A and CPT2 expression were detected by FACS and result examining 6 RA patients and 6 HCs were quantified. **N**-**R** CTLA4 cell surface distribution regulated by CPT2. CD4^+^ T cells isolated from HCs were cultured under Treg condition and transfected with control and CPT2 siRNA on day 3. **N** CPT2 protein level was examined by western blotting. Representative blotting from 3 experiments. **O**-**P** Caveolar expression was detected by FACS and data from 3 experiments were quantified at (**P**). **Q**-**R** CTLA4 cell Surface expression were detected by FACS and data from 3 experiments were quantified at (**R**). All data were presented as the mean ± SEM. *p < 0.05; ***p < 0.001; n.s., non-significance

Lipids play a crucial role in plasma membrane integrity and endocytic processes. To study the metabolic state of lipids in RA Tregs, CD4^+^CD45RO^−^ T cells from patients with RA and age-matched HCs were cultured under Treg-polarizing conditions and stained with the Nile red dye. RA Tregs showed a 1.74-fold higher mean fluorescence intensity (MFI) for lipid accumulation than that observed in HCs (Fig. 3A and B). Consistently, Tregs induced from patients with RA exhibited reduced FAO capability compared to those from HCs, suggesting suppression of FAO pathway in RA Tregs (Fig. 3C and D). In contrast, the expression of PFKFB3, a key regulator of glycolysis, was increased in the RA Tregs (Fig. 3E and F).

CPT1A and CPT2 are the rate-limiting enzymes for fatty acid β-oxidation (Yoon et al. 2022). To assess the levels of CPT subtype in RA T cells, the mRNA and protein levels of CPT1A and CPT2 were determined in Tregs, qPCR and western blotting revealed a dramatically reduction in CPT2 and a moderate decrease in CPT1A expression in RA Tregs compared with that of HCs, which was further confirmed by FACS analysis (Fig. 3G–M, Supplementary Fig. S1).

To determine whether CPT2 deficiency contributed to enhanced CTLA4 endocytosis, CD4^+^ T cells derived from HCs were polarized into Tregs and transfected with CPT2-specific siRNA (Fig. 3N). CPT2 knockdown markedly increased caveolar expression (Fig. 3O and P), thereby decreased CTLA4 surface expression (Fig. 3Q and R), suggesting that CPT2 insufficiency promotes caveolae-mediated CTLA4 endocytosis in RA Tregs.

### Increase in CTLA4 endocytosis associated with impaired UCP2-FAO system

**Fig. 4:**
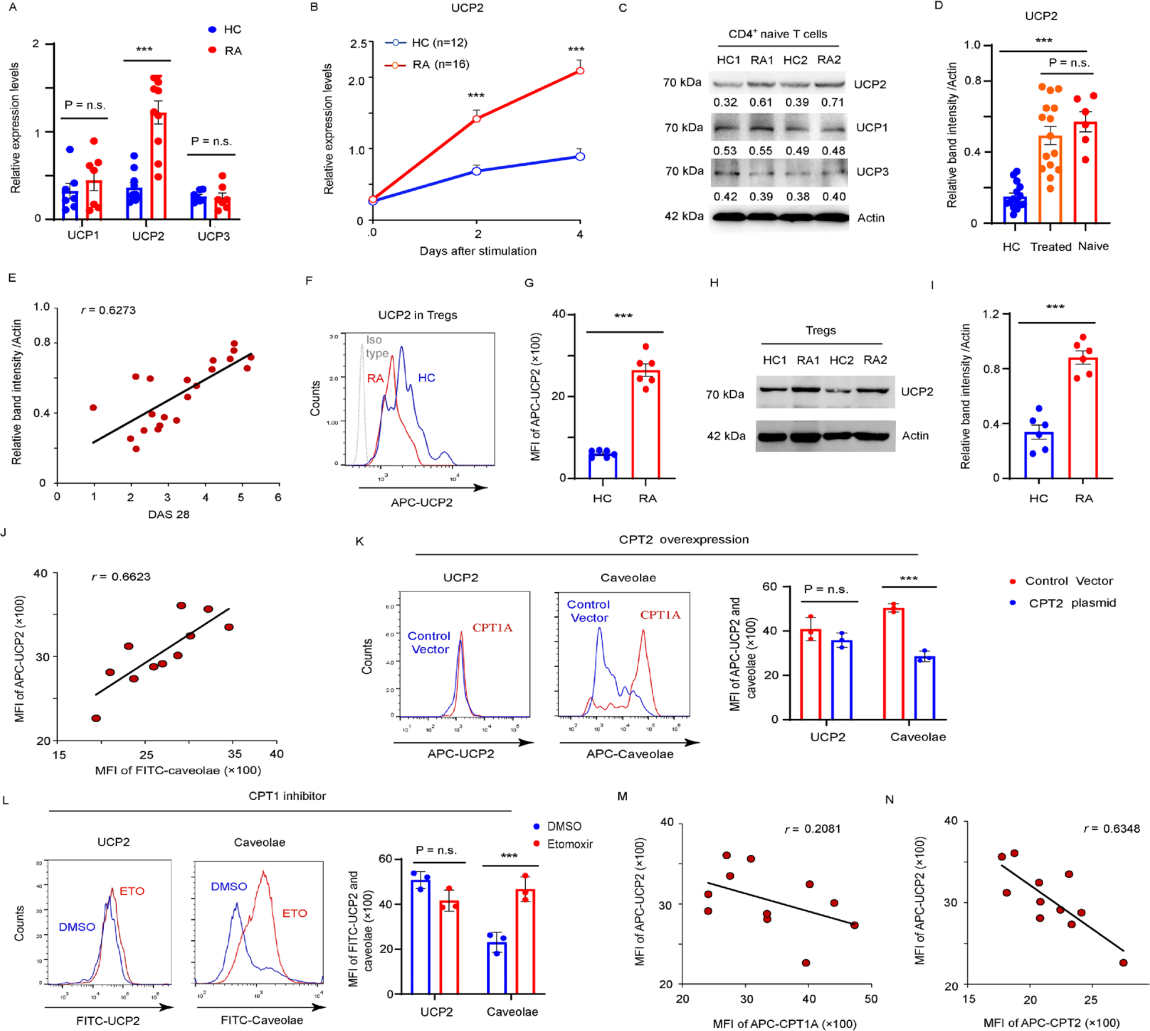
CTLA4 endocytosis increasing is associated with impaired glycolysis-UCP2-FAO system. CD4^+^CD45RO^−^ T cells from RA and HCs were stimulated with anti-CD3/CD28 beads. **A** *UCP1*, *UCP2* and *UCP3* gene expression was quantified by qPCR in CD4^+^ T cells from RA patients and HCs. **B** *UCP2* gene expression on day 0, day 2, and day 4 after anti-CD3/CD28 stimulation. **C** Representative blotting for UCP1, UCP2 and UCP3 expression. **D** UCP2 insufficiency is independent of drug treatment. **E** UCP2 protein expression correlation with disease activity. **F**-**N** CD4^+^CD45RO^−^ T cells from RA patients and HCs were cultured under Treg-polarizing condition. **F**-**G** UCP2 expression in Treg from RA patients and HCs were detected by FACS. The results analyzing 6 RA and 6 HCs were quantified at (**G**). **H**-**I** Representative blotting for UCP2 expression in Tregs. Data analyzing 6 RA and 6 HCs were quantified at (**I**). **J** UCP2 expression in 11 RA patients was assessed for correlation with the caveolar expression. **K** RA-derived Tregs were transfected with GFP-labeled control vectors or GFP-labeled CPT2-expressing plasmids on day 3. The UCP2 and caveolar levels in Tregs were analyzed by FACS. **L** HC-derived Tregs were treated with a FAO inhibitor Etomoxir 20 μM on day 3. The UCP2 and caveolar levels in Tregs were analyzed by FACS. **M**–**N** UCP2 protein expression in Tregs from 11 RA patients was assessed for correlation with the CPT1A and CPT2 expression. All data were presented as the mean ± SEM. ***p < 0.001; n.s., non-significance

Glycolysis and FAO metabolic signaling are bridged by UCP2. However, its role in the generation of CTLA4^int^FOXP3^int^ Tregs in RA remains unclear. To address this issue, UCP1, UCP2, and UCP3 expression in naïve CD4^+^ T cells from RA and HCs were compared following anti-CD3/CD28 stimulation. The results showed that the both gene and protein level of UCP2, but not UCP1 or UCP3, was significantly upregulated in RA CD4 + T cells compared to HCs after TCR stimulation (Fig. 4A–C). To exclude the influence of immunosuppressive therapies on UCP2 induction, patients newly diagnosed with RA who were not receiving disease modifying anti-rheumatic drugs (DMARDs) or corticosteroid treatment were recruited. No significant differences were observed in UCP2 protein expression levels between the treated and untreated patients (Fig. 4D), excluding medications as causative factors inducing UCP2 expression. Notably, UCP2 level positively correlated with disease activity, as indicated by DAS28 (Fig. 4E).

To determine whether UCP2 upregulation was consistent in the Tregs from patients with RA, CD4^+^ T cells from RA and HCs were polarized into Tregs. During Treg differentiation, UCP2 expression was evidently upregulated in RA compared to HCs (Fig. 4F–I). To connect the increase in UCP2 expression with enhanced caveolae-mediated endocytosis in RA Tregs, intracellular UCP2 and caveolar levels in individual Treg cells from RA patients were compared using FACS (Fig. 4J). Higher MFIs of UCP2 were associated with more intense expression of caveolae, suggesting that UCP2 drives caveolae-mediated CTLA4 endocytosis in RA Tregs.

We further examined the links between UCP2 upregulation and impaired FAO metabolism in RA-derived Treg. Tregs induced from HCs were treated with etomoxir, an FAO inhibitor. While the FAO pathway inhibition increased caveolar expression, it did not alter UCP2 levels (Fig. 4K). Similarly, restoring CPT2 expression in RA Tregs only reduced caveolar expression, but had no effect on UCP2 (Fig. 4L), indicating that UCP2 functioned as an upstream suppressor of the FAO pathway. To determine the dominant CPT subtypes regulated by UCP2, intracellular UCP2 level was co-examined with CPT1A and CPT2 levels in individual Treg cells from RA, and a strong negative correlation between UCP2 and CPT2 (*r* = 0.6348) (Fig. 4M), but not CPT1A (*r* = 0.2061) (Fig. 4N), was observed, suggesting that UCP2 might specifically target CPT2 to regulated FAO metabolism.

### UCP2 signaling regulates caveolae-mediated CTLA4 endocytosis through CPT2

**Fig. 5:**
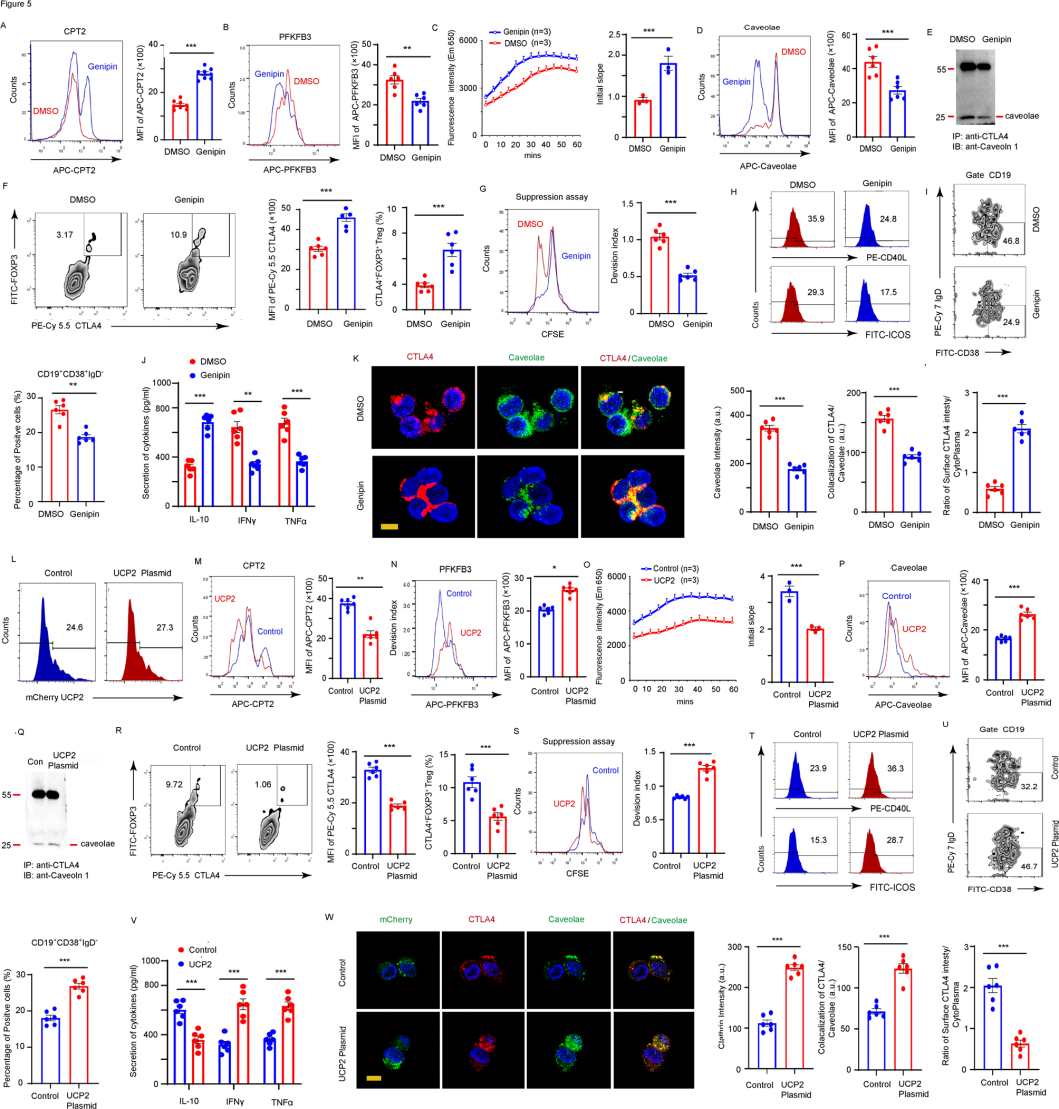
UCP2 signaling regulates caveolae-mediated CTLA4 surface distribution through CPT2. CD4+CD45RO− T cells from RA patients and HCs were cultured under Treg-polarizing condition. **A-K** UCP2 inhibitor treatment. CD4+ T from RA patients were treated with vehicle or UCP2 inhibitor Genipin (25 μM) on day 3 under Treg polarization conditions **A** CPT2 expression was analyzed by FACS. Representative histograms examining 8 RA patients were quantified. **B** PFKPB3 expression was detected by FACS. **C** Time course and rate of increase in oleate-driven FAO assay. Relative FAO rate analyzing 3 RA were quantified. **D** The caveolar levels were analyzed by FACS. **E** Co-immunoprecipitation with CTLA4 antibody followed by blotting with caveolin-1 antibody. **F** The CTLA4 surface expression levels were analyzed by FACS. **G** Proliferation of the CD4+ T cells was analyzed by CFSE dilution. **H** CD40L and ICOS protein levels in CD4+ T cells were analyzed by flow cytometry. **I** A representative density plot of the expression of CD38 and IgD on CD19+ B cells. **J** The production levels of cytokines were measured by ELISA. **K** CTLA4 endocytosis patterns were determined with co-immunostaining CTLA4 with caveolin-1. Fluorescence intensities of caveolae and CTLA4 in the RA Tregs from 3 experiments were quantified. **L-W** UCP2 overexpression. HC-derived CD4+ T cells were transfected with control or mcherry-UCP2 on day 3 under Treg polarization conditions. **L** Transfection efficiency. **M** CPT2 expression was analyzed by FACS. **N** PFKPB3 expression was detected by FACS. **O** Time course and rate of increase in oleate-driven FAO assay. **P** Caveolar levels were analyzed by FACS. **Q** Co-immunoprecipitation for CTLA4 and caveolin-1. **R** CTLA4 surface expression levels were analyzed by FACS. **S** Proliferation of the CD4+ T cells was analyzed by CFSE dilution. **T** CD40L and ICOS levels were analyzed by FACS. **U** The frequencies of CD19+CD38highIgDlow B cells. **V** Expression of cytokines. **W** CTLA4 endocytosis was determined with co-immunostaining CTLA4 with caveolin-1. Bar, 20 μm. All data were presented as the mean ± SEM. ** p < 0.01; *** p < 0.001.

To determine whether the elevated UCP2 expression in RA Tregs drives the enhanced CTLA4 endocytosis via the CPT2 suppression. CD4^+^ T cells isolated from RA were polarized under Treg conditions and treated with Genipin, a selective UCP2 kinase inhibitor. FACS analysis revealed that inhibition of UCP2 enzymic activity substantially upregulated CPT2 while downregulating a key glycolytic enzyme PFKFB3 compared to vehicle-treated Tregs (Fig. 5A and B, Supplementary Fig. S2). This metabolic shift restored FAO in RA Tregs (Fig. 5C), reducing caveolar expression and diminishing the association between CTLA4 and caveolae (Fig. 5D and E). Consequently, the surface expression of CTLA4 was increased, and an elevation in the number of CD25^high^CTLA4^high^FOXP3^high^CD4^high^ Tregs was observed in RA Tregs after Genipin treatment (Fig. 5F). The suppressive properties of Tregs were significantly enhanced after UCP2 inhibition compared to those of the vehicle, as shown by reduced proliferation of CFSE-labeled CD4^+^ T cells (Fig. 5G). Additionally, CD40L and ICOS were decreased in CD4^+^ T cells and the population of CD19^+^CD38^high^IgD^low^ B cell subset emerged with a lower frequency in Tregs treated by Genipin compared to vehicle-treated controls (Fig. 5H and I). These immunomodulatory effects consequently led to reduced effector cytokine secretion and diminished autoantibody production (Fig. 5J and Supplementary Table S2). Moreover, dual-color immunostaining with CTLA4 and caveolin-1 antibodies confirmed reduced levels of caveolar vessels combined with significantly enhanced CTLA4 surface expression in Genipin-treated RA Tregs (Fig. 5K). Similar results were observed when Tregs from patients with RA were transfected with the UCP2 siRNA (Supplementary Fig. S3).

To further validate the role of UCP2 in CTLA4 endocytosis and Treg dysfunction. UCP2 was overexpressed in HC-derived Tregs via plasmid transfection. FACS analysis revealed that both CPT2 expression and FAO metabolism were successfully suppressed by ectopic UCP2 expression, leading to enhanced caveolae-mediated CTLA4 endocytosis (Fig. 5L–Q). Moreover, ectopic expression of UCP2 was sufficient to inhibit the genesis of CD25^high^CTLA4^high^FOXP3^high^CD4^high^ Tregs (Fig. 5R) and impaired their suppressive function against both T and B cells (Fig. 5S–W). These findings indicated that UCP2 promoted the production of dysfunctional CD25^int^CTLA4^int^FOXP3^int^CD4^high^ Tregs by suppressing the CPT2-regulated FAO metabolic pathway.

### UCP2 suppresses CPT2 expression by impairing oxidative signaling and protein acetylation

**Fig. 6:**
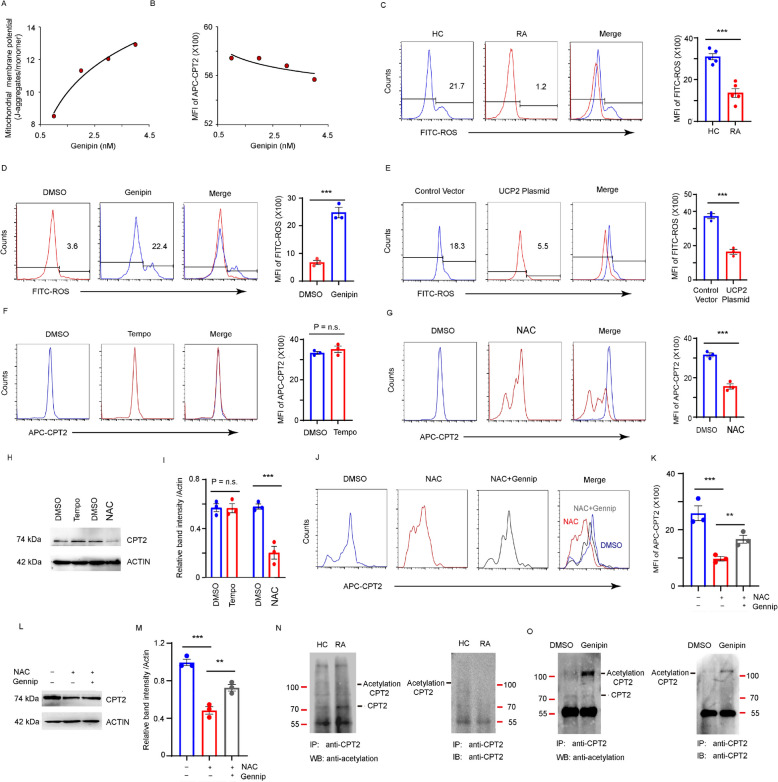
UCP2 suppresses CPT2 expression by impairing oxidative signaling and protein acetylation. CD4^+^CD45RO^−^ T cells from HCs were treated with vehicle or Genipin at indicated dose on day 3 after anti-CD3/CD28 bead stimulation. **A** Correlation of mitochondrial membrane potential (MΦ) with UCP2 activity. MΦ was assessed by FACS. **B** Correlation of CPT2 expression with MΦ. CPT2 expression and MΦ were assessed by FACS. **C-E** CPT2 expression regulated by ROS. **C** ROS levels from RA patients and HCs were measured with H2DCFDA probe on day 3. **D** RA-derived CD4^+^ T cells were treated with Genipin on day 3 and ROS levels were measured after 24 h. **E** HC-derived CD4^+^ T cells were transfected with control and mCherry-UCP2 plasmids on day 3 and ROS levels were measured after 24 h. **F** HC-derived CD4^+^ T cells were treated with vehicle or TEMPO (20 μM) on day 3 for 24 h. CPT2 levels were measured by FACS. **G** HC-derived CD4^+^ T cells were treated with vehicle or NAC 20 μM on day 3 for 24 h. CPT2 levels were measured by FACS. **H**-**I** CPT2 expression after TEMPO and NAC treatment was measured by Western blotting and quantified at (**I**). **J**-**K** HC-derived CD4^+^ T cells were treated with vehicle, NAC 20 mM, or NAC and Genipin 25 μM on day 3 for 24 h. **J** CPT2 levels were measured by FACS and quantified at (**K**). **L**-**M** CPT2 protein expression after NAC 20 mM, or NAC and Genipin treatment were measured by Western blotting. **N** CPT2 acetylation was determined by a Co-immunoprecipitation assay with anti-CPT2 followed by immunoblotting with anti-acetylated-lysine antibody. **O** RA-derived CD4^+^ T cells were treated with Genipin and CPT2 acetylation were measured by Co-immunoprecipitation. All data were presented as the mean ± SEM. **p < 0.01; ***p < 0.001; n.s., non-significance

UCP2 and CPT2 co-exist in the inner mitochondrial membrane, suggesting a potential interaction. To investigate the underlying mechanism by which UCP2 regulates CPT2 expression, CD4^+^CD45RO^−^ T cells isolated from patients with RA were activated with anti-CD3/CD28 beads and treated with Genipin. A dose-dependent increase in mitochondrial membrane potential (mΦ) following UCP2 inhibition was observed, indicating that UCP2 performed uncoupling functions upon T cell activation in RA (Fig. 6A). However, the kinetics of CPT2 expression were not strongly associated with the changes in mΦ (Fig. 6B). Therefore, the regulation of CPT2 expression by UCP2 was unlikely to be an immediate consequence of the mitochondrial uncoupling function of UCP2.

The intracellular ROS levels in CD4^+^ T cells from patients with RA were nearly 50% lower than those in HCs following TCR stimulation, as measured by the ROS probe H_2_DCFDA (Yang et al. 2016) (Fig. 6C). To elucidate whether high UCP2 level was responsible for the reduced ROS levels in RA T cells, UCP2-expressing plasmid were transfected into HC-derived CD4^+^ T cells, and ectopic expression of UCP2 resulted in reduced ROS levels compared to the vehicle controls (Fig. 6D). Furthermore, UCP2 inhibition in RA-derived CD4^+^ T cells restored ROS production (Fig. 6E). These findings suggested that UCP2 suppress ROS genesis in RA T cells.

To further examine whether UCP2-mediated CPT2 regulation involved oxidative signaling pathway, HC-derived CD4^+^ T cells were treated with two ROS scavengers that Suppress ROS production via different mechanisms. 2,2,6,6-Tetramethylpiperidinooxy (TEMPO), which converts toxic superoxide molecules to hydrogen peroxide, whereas N-acetyl-L-cysteine (NAC) serves as a precursor of glutathione. FACS analysis and western blotting revealed that the NAC, but not TEMPO, reduced CPT2 expression (Fig. 6F-I), implicating mitochondrial glutathione metabolism in CPT2 regulation. Moreover, Genipin partially restored CPT2 levels in NAC-treated RA Tregs (Fig. 6J–M), further supporting that UCP2 may regulate CPT2 expression through glutathione-mediated ROS signaling.

Acetylation plays a key role in maintaining protein stability (Fan et al. 2022), and CPT2 protein stability was modulated through acetylation modification (Supplementary Fig. S4). To examine whether CPT2 acetylation was intact in RA-derived CD4^+^ T cells, CPT2 acetylation levels was measured via immunoprecipitation using a CPT2 antibody followed by a reaction with an anti-acetylated-lysine. A substantially weaker band intensity of acetylated CPT2 was observed in RA T cells compared to those from HCs (Fig. 6N). Furthermore, UCP2 inhibition substantially increased the expression of acetylated–CPT2 (Fig. 6O), indicating that UCP2 suppressed CPT2 expression by impairing its acetylation in RA.

### UCP2 expression in RA T cells induces synovial tissue inflammation

**Fig. 7:**
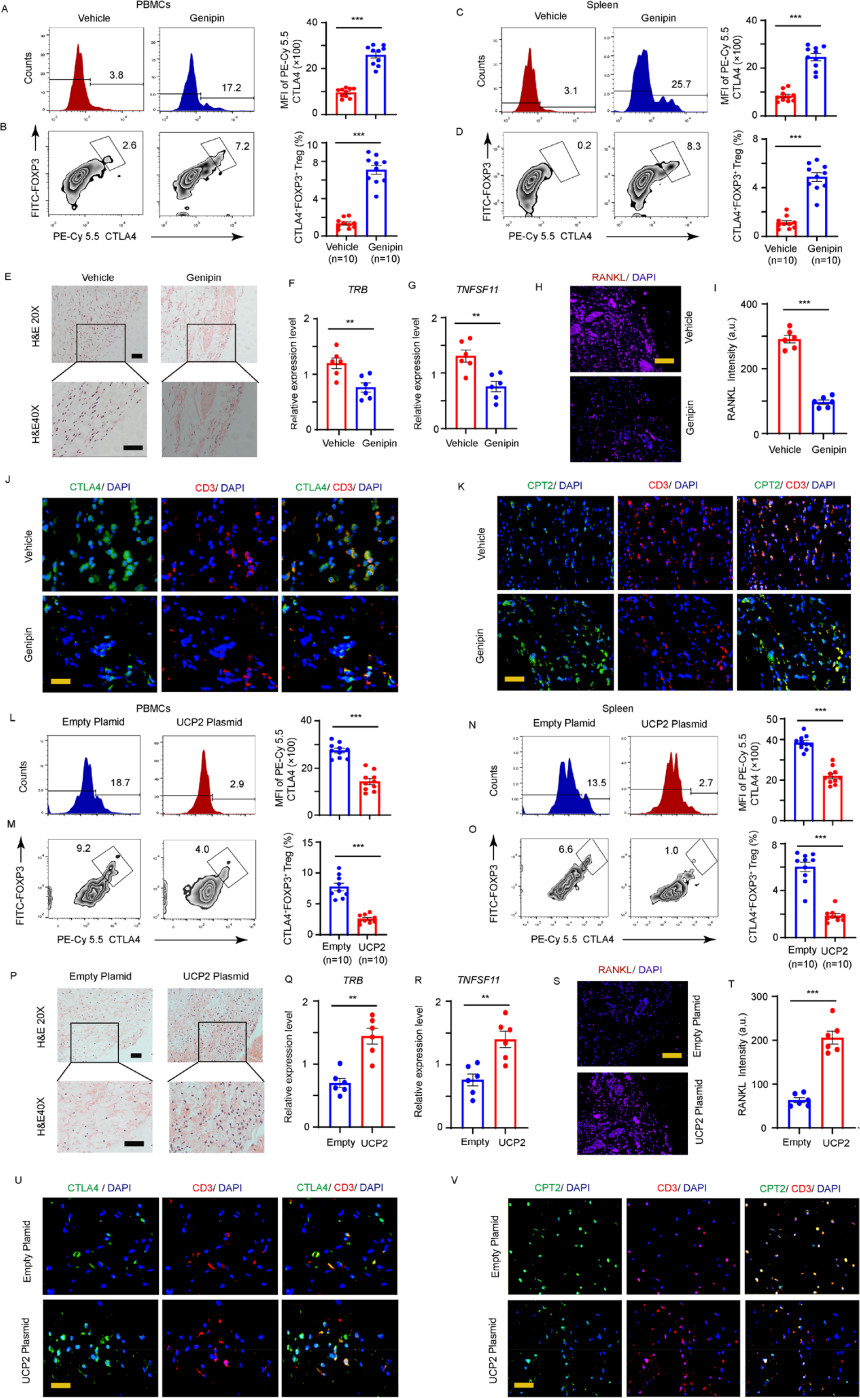
UCP2 expression in RA T-cells induces synovial tissue inflammation. Pairs of NSG mice were engrafted with synovial tissue from RA patients, and CD45RO^−^ PBMCs from HCs were transferred to the chimeric mice. The mice were divided into two groups, vehicle (DMSO) and Genipie groups. **A**-**B** CTLA4 surface expression was detected by FACS in the CD4^+^ T cells from PBMCs. **C**-**D** CTLA4 surface expression was detected by FACS in the CD4^+^ T cells from spleen. **E** Representative H&E images. **F**-**G** The intensity of synovial inflammation was compared by qPCR to assess *TRB* and *TNFSF11* gene expression in each group. **H**-**I** RANKL staining. **J** Representative images of anti-CD3 (red) and anti-CTLA4 staining (green) of the vehicle and UCP2 inhibitor-treated mice. Bar, 20 μm. **K** Co-immunostaining of CPT2 (green) and CD3 (red). Representative images were from one of three synovial tissue from each group. Bar, 20 μm. **L**-**V** UCP2 overexpression. CD45RO^−^ PBMCs from RA were transfected with either control or mCherry-UCP2 plasmids and adoptively transferred to the chimeric mice. **L**-**M** CTLA4 surface expression was detected by FACS in the CD4^+^ T cells from PBMCs. **N**–**O** CTLA4 surface expression were detected by FACS in the CD4.^+^ T cells from spleen. **P** Representative H&E images. **Q**-**R** *TRB* and *TNFSF11* gene expression was compared by qPCR. **S**-**T** RANKL staining. **U** Representative images of anti-CD3 (red) and anti-CTLA4 staining (green). Bar, 20 μm. **V** Co-immunostaining of CPT2 (green) and CD3 (red). Bar, 20 μm. All data were presented as the mean ± SEM. **p < 0.01; ***p < 0.001

To investigate the process by which T cells initiate and drive synovitis, we employed a human SCID chimeric mouse model by transplanting synovial tissues from patients with RA into immunodeficient mice, and the CD45RO^−^ PBMCs from healthy donors were adoptively injected into the chimeric mice. Those mice were subsequently treated with the Genipin. Inhibition of UCP2 activity resulted in a significant increase in the production of CD25^high^CTLA4^high^FOXP3^high^CD4^high^Tregs in both PBMCs and the spleens (Fig. 7A–D). H&E staining showed reduced immune cell infiltration in the synovial tissues (Fig. 7E), supported by downregulated expression of the T-cell receptor beta locus (*TRB*) and *TNFSF11* (Fig. 7F and G). Additionally, the protein expression levels of osteoclastogenic ligand RANKL, a key mediator of rheumatoid bone destruction, was nearly threefold lower in the Genipin-treated synovial tissues (Fig. 7H and I).

To further elucidate the possible link between FAO metabolism and Treg differentiation, T cell subsets were quantified by dual-color immunofluorescence staining using CTLA4 or CPT2 combined with CD3 antibodies. After treatment with a UCP2 inhibitor, RA T cells exhibited a notable increase in CTLA4 intensity on the cell surface, and the increase in CTLA4^high^ T cells was associated with enhanced CPT2 expression in synovial sections from mice treated with the Genipin compared to those treated with the vehicle (Fig. 7J and K). Furthermore, Tregs enrichment was confirmed by increased level of inhibitory cytokine IL-10 and TGFβ, combined with decreased pro-inflammatory cytokine IL-6 and TNFα production (Supplementary Fig. S5A and B).

To determine whether the ectopic expression of UCP2 in CD4^+^ T cells was sufficient to induce arthritogenic effects, UCP2 was overexpressed in CD4^+^ T cells from HCs prior to adoptive transfer. UCP2 overexpression reduced the frequency of CD25^high^CTLA4^high^FOXP3^high^CD4^high^ Tregs in PBMCs and splenic tissues (Fig. 7L–O). Consequently, immune cell infiltration was strongly induced co-occurred with elevated RANKL levels in engrafted synovial tissues (Fig. 7P–T). Mechanistically, UCP2 overexpression inhibited CPT2 expression, which subsequently attenuated CTLA4 accumulation in transferred T cells (Fig. 7U and V). As a result of Treg recovery, synovial tissue exhibited elevated production of IL-10 and TGFβ (Supplementary Fig. S5C and D). These results verified that UCP2 drive disease-associated Treg genesis by suppressing the FAO pathway and impairing CTLA4 surface distribution.

## Discussion

As a critical inhibitory checkpoint receptor, CTLA4 is constitutively expressed on activated CD^+^ T cells. CTLA4 have identified as a critical mediator of the suppressive effects of Tregs (Flores-Borja et al. 2008; Wing et al. 2008). In patients with RA, the predominant Treg population is characterized by CTLA4^int^FOXP3^int^ T cells with impaired CTLA4 cell surface expression, rather than the canonical CTLA4^high^FOXP3^high^ Treg population, which is known for robustly suppressing effector T cells. Notably, driving CTLA4 to the cell membrane of RA Tregs using PMA restored their suppressive function, and this effect was reversed by CTLA4 blockade (Alvarez-Quiroga et al. 2011). These evidence suggests that the deficient CTLA4 membrane distribution underlies Treg dysfunction in RA (Flores-Borja et al. 2008). Although CTLA4 can be efficiently transported to the cell surface, it is rapidly endocytosed into the cytoplasm, resulting in its predominant intracellular localization. Following TCR stimulation, however, the rate of CTLA4 endocytosis decreases, allowing CTLA4 to remain on the cell surface and prevent T cell overactivation. Our study revealed that the increased rate of CTLA4 internalization in RA Tregs was mediated by caveolae-dominated endocytosis. Interestingly, other immunoinhibitory molecules such as LAG3 and PD-1, were overexpressed on the surface of RA Tregs (Chen et al. 2016; Nakachi et al. 2017; Koohini et al. 2018), potentially compensating for the reduced CTLA4 surface expression to maintain an immunoinhibitory state. A critical question arises regarding the role of CTLA4^int^FOXP3^int^ Tregs in the RA pathogenesis. Our study demonstrated that CTLA4^int^FOXP3^int^ Tregs exhibited reduced suppressive capability for both effector T cells and B cells, likely contributing to the autoimmune genesis in RA.

Previous studies have reported that defects in lipid metabolism in RA T cells would lead to excessive intracellular lipid storage and altered cell surface mobility (Shen et al. 2017). Consistently, our study indicated that the FAO pathway, responsible for lipid-acid consumption, was suppressed in RA Tregs. Caveolae-mediated endocytosis acted as a negative responder of FAO metabolism. Therefore, the attenuation of FAO signaling in RA Tregs upregulated caveolar expression, subsequently causing CTLA4 internalization. A reduction in surface expression of CTLA4 was observed in both Tregs and conventional T cells. However, given that Tregs primarily rely on FAO for energy and require high FAO metabolic activity, impaired FAO metabolism may play a central role in Treg dysfunction in RA.

T cell differentiation is tightly regulated by cellular metabolism. For instance, differentiated Th17 cells exhibit enhanced glycolytic activity, whereas Tregs prefer FAO for their energy needs (Maciolek et al. 2014; Zhang et al. 2019). This suggests the existence of a metabolic state-sensing system that orchestrates the switch between glycolysis and the FAO pathway based on T cell functional requirements. Our study showed that the suppression of FAO, combined with elevated glycolytic activity in RA Tregs, contributed to their diminished suppression functions. UCP2, a regulator of cellular energy homeostasis, modulates glucose and fatty acid metabolism (Rousset et al. 2004; Pecqueur et al. 2008; Rangarajan et al. 2022; Vozza et al. 2014). Here, we identified a significant increase in UCP2 expression in RA Tregs, raising the question of whether the imbalance in glycolysis and FAO pathways in RA was linked to UCP2. Our findings demonstrated that the increased glycolysis was associated with elevated UCP2 expression in RA Tregs, consistent with the reports that UCP2 is a key indicator of the glycolytic pathway, allowing cells to remain at the glycolytic level in aerobic environments (Bouillaud 2009). Furthermore, inhibiting UCP2 activity restored FAO metabolism, whereas UCP2 expression remained unaffected by FAO inhibition, indicating that UCP2 acted as an upstream suppressor of the FAO pathway.

Further studies have suggested that UCP2 attenuated FAO metabolism by targeting CPT2 rather than CPT1A. The primary effects of UCP2 include protein uncoupling, ROS regulation, and protein post-translational modification (Palanisamy et al. 2014; Giardina et al. 2008; Zhao et al. 2019). Although UCP2 and CPT2 co-existed in the mitochondrial membrane, our results clearly showed that the UCP2-mediated regulation of CPT2 expression was not closely associated with its uncoupling activity. We observed that increased UCP2 expression contributed to lower ROS levels in CD4^+^ T cells, and decreased ROS levels reduced CPT2 expression. Moreover, CPT2 expression was effectively downregulated by the glutamine precursor NAC, but not by the superoxide scavenger TEMPO. These findings align with the reports that UCP2 regulates mitochondrial respiration through glutamine-dependent oxidative metabolism (Vozza et al. 2014; Sancerni et al. 2022; Raho et al. 2020). A recent study reported that the UCP2 regulates oxidative stress by upregulating the expression of the deacetylase SIRT3 (Zhao et al. 2019). Our study revealed decreased CPT2 acetylation levels and protein concentration in RA T cells compared to HCs, suggesting that UCP2-mediated regulation of CPT2 expression may depend on the CPT2 acetylation.

Using a human synovium–NSG mouse model combined with human T-cell injection (Hwang et al. 2015), we observed that the UCP2-FAO-caveolae-CTLA4 axis was strongly associated with clinical RA atherogenesis. Inhibition of UCP2 activity decreased caveolae-mediated CTLA4 endocytosis and enhanced Treg differentiation, thereby suppressing the production of pro-arthritic T cells in the synovial microenvironment. Conversely, rescuing UCP2 expression promoted caveolae-mediated CTLA4 endocytosis, attenuated Treg differentiation, and significantly enhanced inflammatory immune responses in synovial tissues. These results underscored the importance of UCP2 in regulating CTLA4 cellular localization and its impact on Treg function during the onset of synovial inflammation, illustrating that modulating UCP2 activity can influence the balance between pro-inflammatory and anti-inflammatory T cell populations in RA synovial tissues.

## Conclusion

Our findings demonstrated that elevated expression of UCP2 in RA Tregs leads to the reduced FAO and enhanced caveolae-mediated CTLA4 endocytosis, promoting the production of CTLA4^int^FOXP3^int^ Treg. Given the pivotal role of Treg dysfunction in the pathogenesis of RA autoimmunity, targeting UCP2 to restore Treg function may represent a promising therapeutic strategy for preventing RA autoimmunity.

## Supplementary Information

Supplementary material 1.

Supplementary material 2. Supplemental Fig. S1. Fatty acid oxidation and endocytosis pathway in CD4^+^T cells. Supplemental Fig. S2. UCP2 mild control CPT1A expression. Supplemental Fig. S3. UCP2 knockdown increased CTLA4 surface distribution through upregulation CPT2. Supplemental Fig. S4. CPT2 protein modified by acetylation. Supplemental Fig. S5. UCP2 controls the arthritogenic effect of T cells. Supplemental Fig. S6. FMO control for flow cytometer. Supplemental Table S1. Primers sequence for qPCR assays. Supplemental Table S2. Autoantibody Panel. Supplemental Table S3. Reagents.

## Data Availability

No datasets were generated or analysed during the current study.

## Abbreviations

Treg: Regulatory T cell
RA: Rheumatoid arthritis
FOXP3: Fork-head box protein 3
CTLA4: Cytotoxic T lymphocyte antigen 4
UCP2: Uncoupling protein 2
FAO: Fatty acid β-oxidation
PBMCs: Peripheral blood mononuclear cells
SFs: Synovial fluids
MFI: Mean fluorescence intensity
FACS: Fluorescence activated cell sorting
TCR: T cell receptor
CLTC: Clathrin heavy chain
CLTA: Clathrin light chain
CPT2: Carnitine palmitoyltransferase 2
PFKFB3: 6-Phosphofructo-2-kinase/fructose-2,6-biphosphatase 3
SIRT3: Sirtuin 3
