# Supplementary figures and images for: Dysfunctional glycolysis-UCP2-fatty acid oxidation promotes CTLA4intFOXP3int regulatory T-cell production in rheumatoid arthritis
Source: Mol Med. 2025 Oct 9;31:310. doi: 10.1186/s10020-025-01372-6 (PMC12512393; doi:10.1186/s10020-025-01372-6)

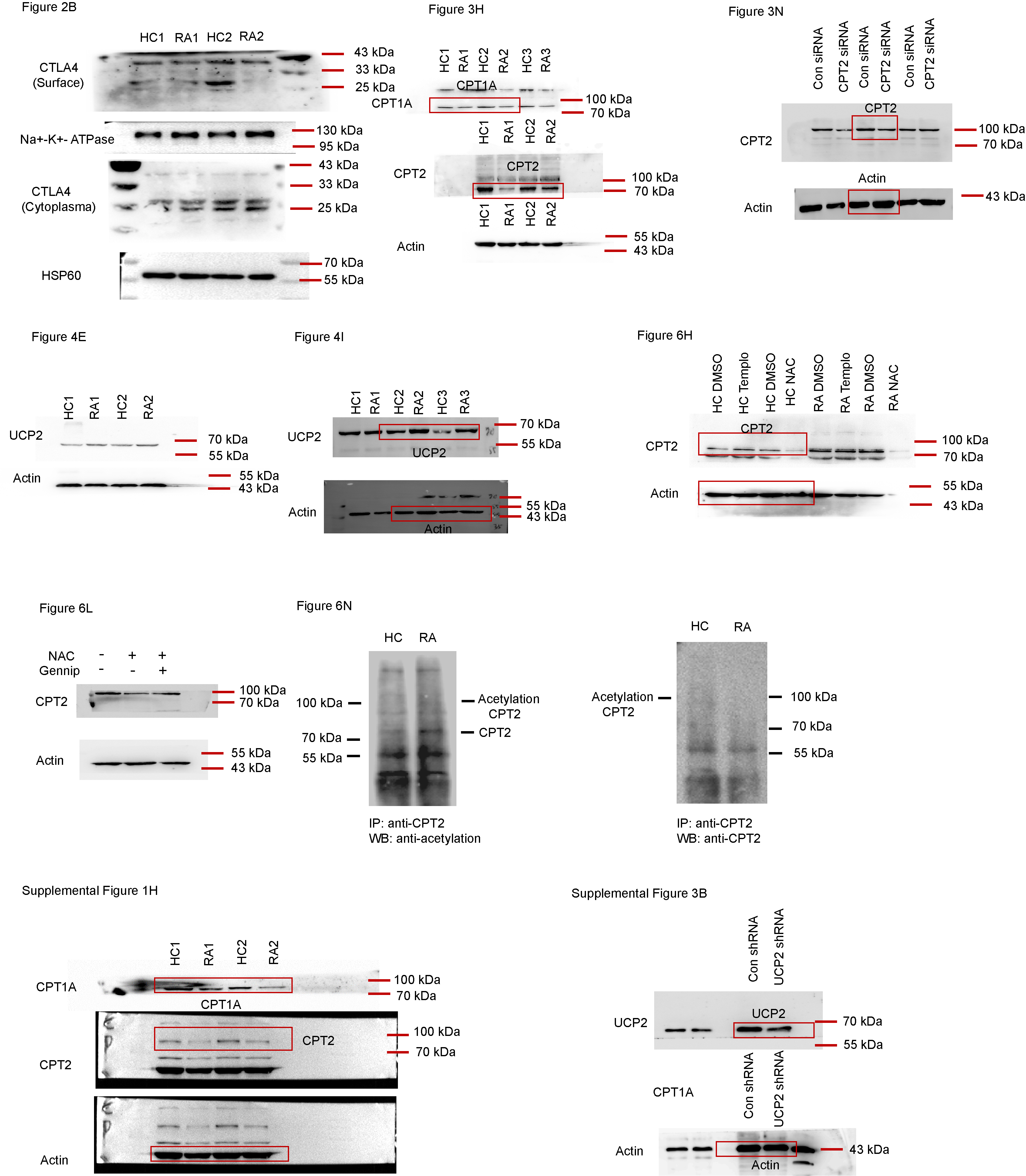

Supplement: Supplementary file 1 — Supplementary material 1. [file 10020_2025_1372_MOESM1_ESM.tif]
