# Supplementary material for: Dysfunctional glycolysis-UCP2-fatty acid oxidation promotes CTLA4intFOXP3int regulatory T-cell production in rheumatoid arthritis
Source: Mol Med. 2025 Oct 9;31:310. doi: 10.1186/s10020-025-01372-6 (PMC12512393; doi:10.1186/s10020-025-01372-6)
Supplement: Supplementary file 2 — Supplementary material 2. Supplemental Fig. S1. Fatty acid oxidation and endocytosis pathway in CD4+T cells. Supplemental Fig. S2. UCP2 mild control CPT1A expression. Supplemental Fig. S3. UCP2 knockdown increased CTLA4 surface distribution through upregulation CPT2. Supplemental Fig. S4. CPT2 protein modified by acetylation. Supplemental Fig. S5. UCP2 controls the arthritogenic effect of T cells. Supplemental Fig. S6. FMO control for flow cytometer. Supplemental Table S1. Primers sequence for qPCR assays. Supplemental Table S2. Autoantibody Panel. Supplemental Table S3. Reagents. [file 10020_2025_1372_MOESM2_ESM.docx]

**Supplementary Information**

**Dysfunctional Glycolysis-UCP2-Fatty Acid Oxidation Promotes CTLA4^int^FOXP3^int^ Regulatory T-Cell Production in Rheumatoid Arthritis**

**Running Title: UCP2 Promotes CTLA4^int^ Treg Production in RA**

Jiawen Han^1†^, Zhongyang Zhou^1†^, Hongxia Wang^2,3†^, Yuxin Chen^4†^, Wuguo Li^1^, Meiqin Dai^1^, Jing Bian^1^, Erming Zhao^1^, Jiaying He^1^, Xinyao Zhang^5^, Huanfa Yi^6^, Lan Shao^1^*

1The Center for Translational Medicine, The First Affiliated Hospital, Sun Yat-Sen University, Guangzhou, P.R. China 510080; 2Laboratory Medicine Center, Nanfang Hospital, Southern Medical University, Guangzhou, P.R. China 510515; 3Department of Rheumatology, Nanfang Hospital, Southern Medical University, Guangzhou, P.R. China 510515; 4Department of Laboratory Medicine, Nanjing Drum Tower Hospital, Affiliated Hospital of Medical School, Nanjing University, Nanjing, P.R. China 210008; 5Department of Urology, Henan Cancer Hospital, Zhengzhou, P.R. China 450008; 6Central Laboratory, The First Hospital of Jilin University, Changchun, P.R. China 130061;

^*^Corresponding authors:

1 Dr. Lan Shao, The Center for Translational Medicine, The First Affiliated Hospital, Sun Yat-Sen University, Guangzhou, P.R. China 510080; Tel: 0086-20-87755766: Fax: 0086-20-87755766; E-mail: [shaolan@mail.sysu.edu.cn](mailto:shaolan@mail.sysu.edu.cn)

^†^ These authors contributed equally to this work.

**Supplemental Figures S1-6 and Supplemental Table 1-3**

Supplemental Fig. S1. Fatty acid oxidation and endocytosis pathway in CD4^+^ T cells

Supplemental Fig. S2. UCP2 mild control CPT1A expression

Supplemental Fig. S3. UCP2 knockdown increased CTLA4 surface distribution

through upregulation CPT2

Supplemental Fig. S4. CPT2 protein modified by acetylation

Supplemental Fig. S5. UCP2 controls the arthritogenic effect of T cells

Supplemental Fig. S6. FMO control for flow cytometer

Supplemental Table S1. Primers sequence for qPCR assays

Supplemental Table S2. Autoantibody Panel

Supplemental Table S3. Reagents

**Supplementary Material and Method**

1. **Deacetylase Inhibition and Activation Assays**

RA-derived CD4⁺ T cells were stimulated with anti-CD3/CD28-coupled beads and cultured with IL-2 (10 ng/ml) and TGF-β1 (10 ng/ml) to induce Treg differentiation. On day 3 after stimulation, the cells were treated with either the deacetylase inhibitor 3-(1H-1,2,3-triazol-4-yl) pyridine (3TYP) (20 μM; #120241-79-4, Macklin, China) or the SIRT activator Nicotinamide riboside (1 mM; #1341-23-7, Macklin). The cells were harvested 24 hours post-treatment.

1. **Anti-Streptolysin O (ASO)**

ASO levels in cell culture Surprenant were quantified using the Siemens BNII specific protein analyzer (Siemens, Germany). Specifically, samples containing human ASO were incubated with polystyrene particles coated with streptolysin O, resulting in specific antigen-antibody complexes formation. The scattered light intensity of this immune complex directly correlating to the ASO concentration. The content of ASO in the sample was determined based on standard curves generated from known standards.

1. **Rheumatoid Factor (RF)**

Rheumatoid Factor (RF) was measured using the Siemens BNII specific protein analyzer (Siemens, Germany). Specifically, polystyrene particles coated with immunocomplexes (gamma globulin/sheep anti-human gamma globulin) were mixed with samples, leading to the agglutination of particles. Consequently, light passing through the mixture scattered, and the intensity of the scattered light was proportional to the concentration of RF. The content of RF in the samples was determined based on known standards.

1. **C-Reactive Protein (CRP)**

C-reactive protein (CRP) was detected using the Roche Cobas c7500 fully automated biochemistry analyzer (Roche, Basel, Switzerland). The measurement employed a particle-enhanced turbidimetric immunoassay method. The content of CRF in the sample was determined based on standard curves generated from known standards.

1. **Anti-β₂GP1-IgG/IgM Antibody**

Anti-β₂GP1-IgG/IgM antibodies were tested using the Alegria fully automated immunoassay system (Xiupeng Biotechnology Development Co., Ltd. Tianjin, China). A magnetic particle coated with human β₂GP1 were used to captured anti-β₂GP1 antibodies in the samples. Following incubation, magnetic separation, an isoluminol-labeled anti-human IgG/IgM tracer antibody was introduced, and a trigger solution was then added to induce chemiluminescence. The resulting light emission was measured in Relative Light Units (RLU), with the RLU value being proportional to the concentration of anti-β₂GP1 antibody.

1. **Anti-CCP Antibody (Anti-Cyclic Citrullinated Peptide Antibody)**

Anti-CCP antibody was analyzed using the Maglumi X8 fully automated chemiluminescence immunoanalyzer (New Industries Biomedical Engineering Co., Ltd. Shenzhen, China). CCP-coated magnetic beads were employed to capture anti-CCP antibodies. After the washing step, the ABEI-labeled mouse anti-human IgG and substrate were added, and the resulting chemiluminescent signal was measured, which was proportional to the concentration of anti-CCP antibody.

**
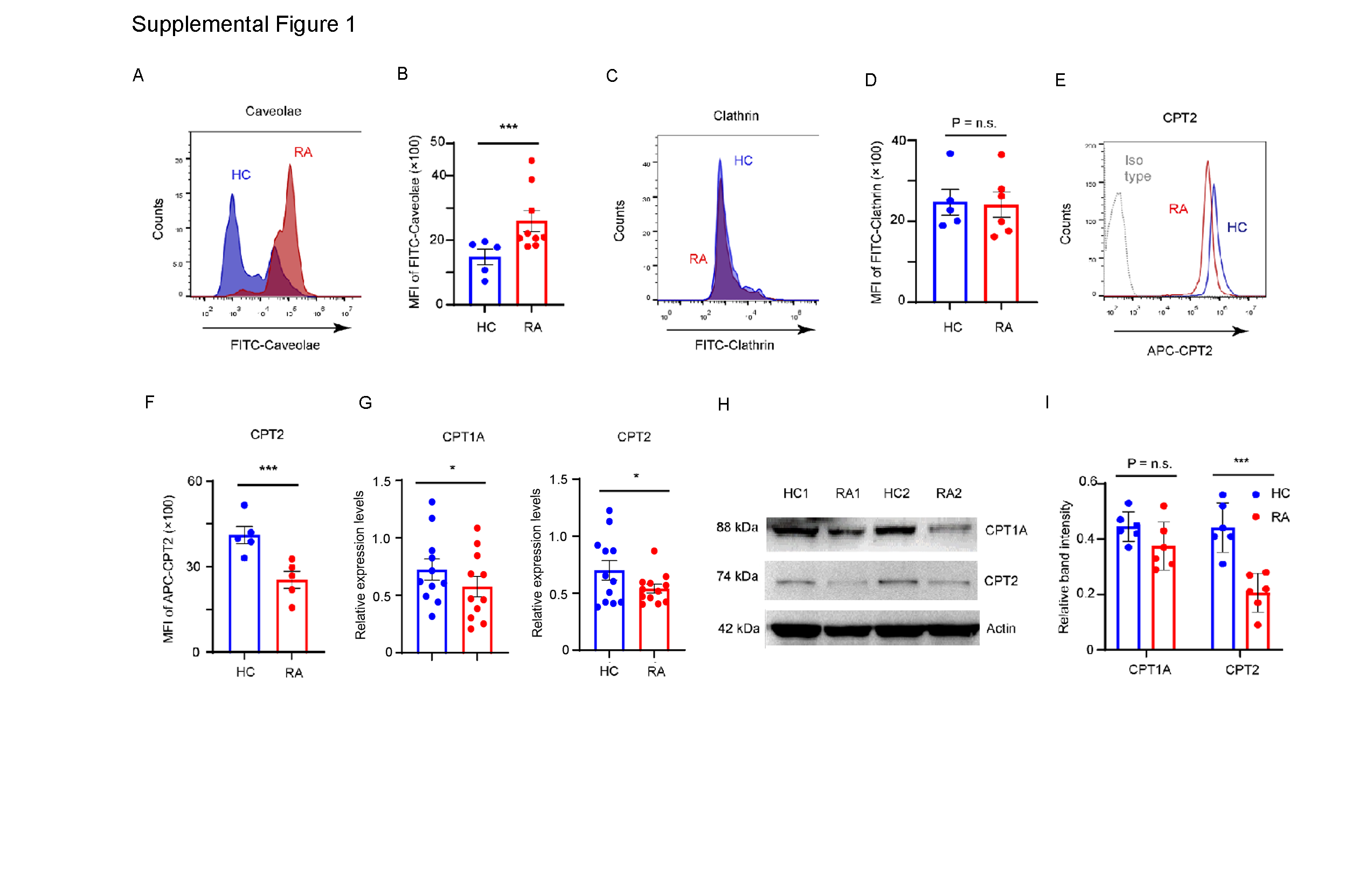
**

**Supplemental Figure S1.** **Fatty acid oxidation and endocytosis pathway in CD4^+^ T cells.** CD4^+^CD45RO^−^ T cells from RA and HCs were stimulated with anti-CD3/CD28 beads. (A-B) Caveolar expression were detected by FACS and results examined 9 RA patients and 5 HCs were quantified at B. (C-D) Clathrin expression was detected by FACS and results examined 6 RA patients and 5 HCs were quantified. (E-F) CPT2 expression was detected by FACS and results examined 5 RA patients and 5 HCs were quantified. (G) *CPT1A and CPT2* gene expression was quantified by qPCR in CD4^+^ T cells from RA patients (n=12) and HCs (n=12). (H-I) Representative blotting for CPT1A and CPT2 protein expression. Results analyzing 6 RA patients and 6 HCs were quantified at I. All data were presented as the mean ± SEM. *p < 0.05; ***p < 0.001; n.s., non-significance.


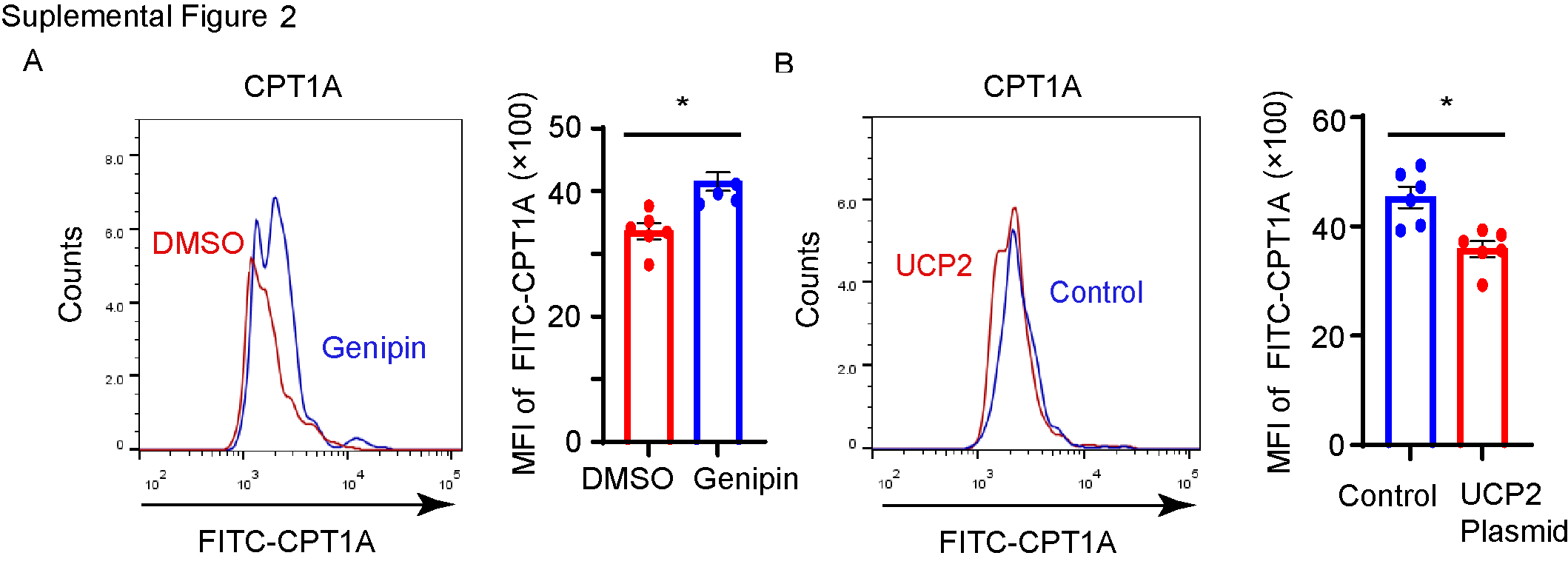


**Supplemental Figure S2.** **UCP2 regulated CPT1A expression.** CD4^+^CD45RO^−^ T cells from RA patients and HCs were cultured under Treg-polarizing condition. (A) Tregs from RA patients were treated with vehicle or UCP2 inhibitor Genipin (25 μM) on day 5. CPT1A expression was analyzed by FACS. Representative histograms examining 6 RA patients were quantified. (B) Tregs from HCs (n=6) were transfected with control or mcherry-UCP2. CPT1A expression was analyzed by FACS. Representative histograms examining 6 RA patients were quantified. All data were presented as the mean ± SEM. *p < 0.05.

**
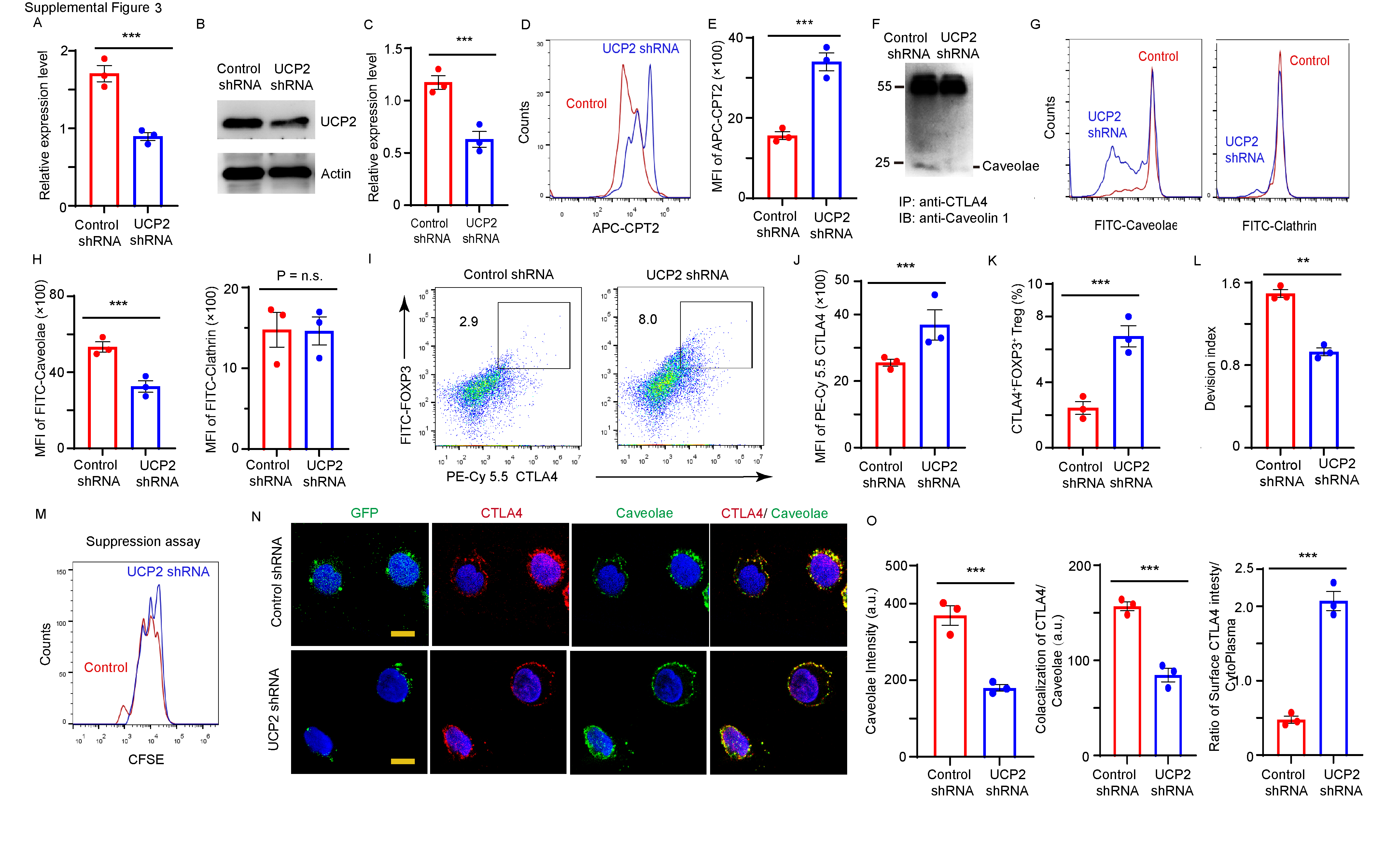
**

**Supplemental Figure S3.** **UCP2 knockdown increased CTLA4 surface distribution through upregulation CPT2.** CD4^+^ T cells from RA patients were transfected with pLV-mCherry-U6>*UCP2*_shRNA or with pLV-mCherry-U6 >Scramble_shRNA on day 3 after anti-CD3/CD28 bead stimulation. (A) *UCP2* transcript levels were quantified by qPCR. (B-C) Protein expression of UCP2 were detected by western blotting and qualified at C. (D-E) CPT2 expression were analyzed by FACS. Representative histograms examining 3 RA patients were quantified at E. (F) Co-immunoprecipitation for CTLA4 and caveolin-1. (G-H) The caveolar and clathrin levels were analyzed by FACS. (I-M) T cell differentiation. CD4^+^CD45RO^−^ T cells were cultured under Treg-polarizing conditions (I-K) CTLA4 surface expression was quantified at I and percentages of Tregs for 3 experiments examining were quantified at J. (L-M) Proliferation of the CD4^+^ T cells was analyzed by CFSE dilution. (N-O) CTLA4 endocytosis patterns were determined with co-immunostaining with anti-caveolin-1. (N) A representative image is shown. Bar, 20 μm. (O) Fluorescence intensities of caveolae and CTLA4 in the CD4^+^ T cells. All data were presented as the mean ± SEM. **p < 0.01; ***p < 0.001. n.s., non-significance.


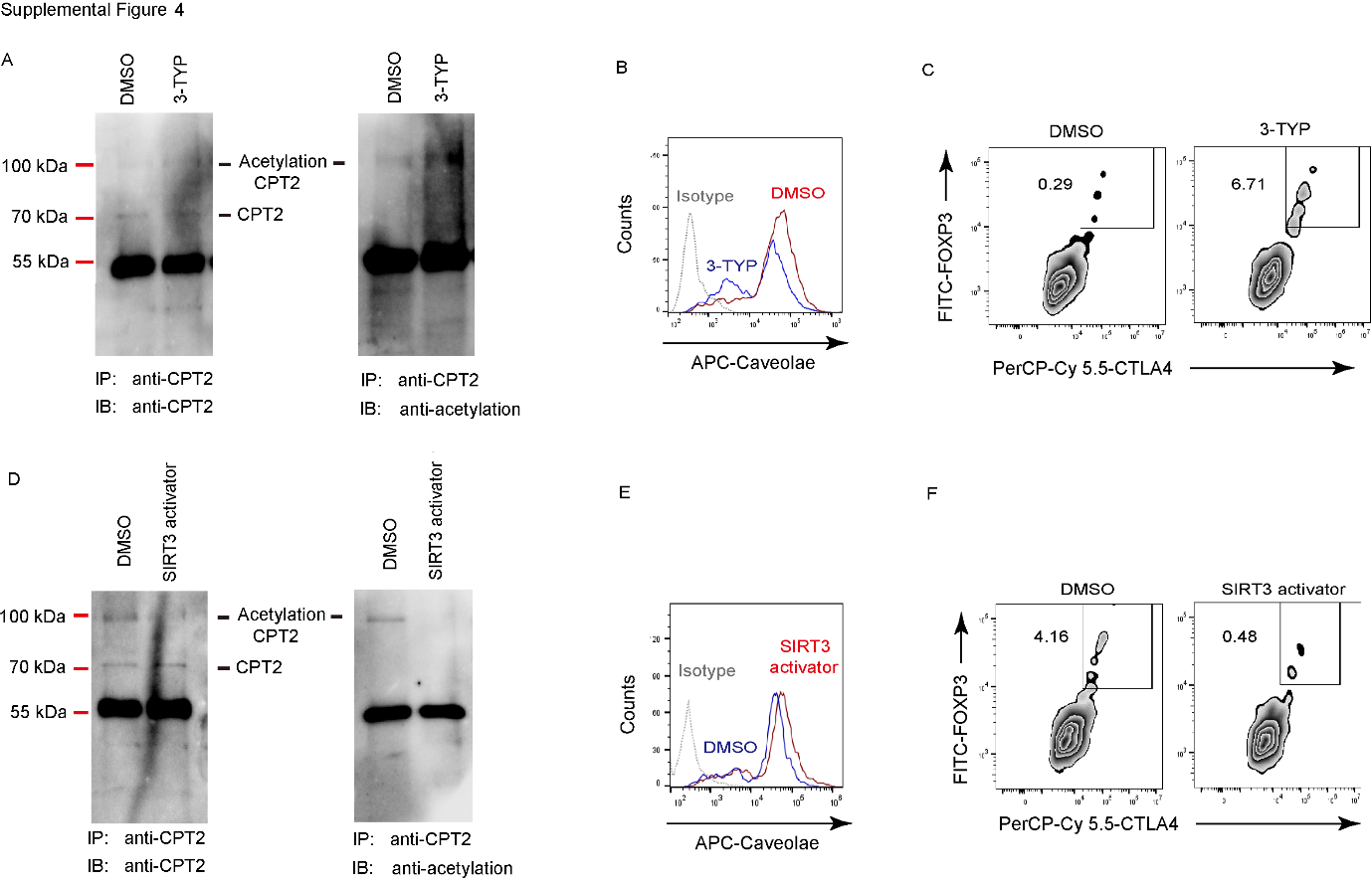


**Supplemental Figure S4. CPT2 protein modified by acetylation.** (A-C) RA-derived CD4^+^ T cells were cultured under Treg-polarizing condition. Deacetylase inhibitor 3-(1H-1,2,3-triazol-4-yl) pyridine (3TYP) 20 μM was added on day 3 for 24 h. (A) CPT2 acetylation was determined by a Co-immunoprecipitation assay with anti-CPT2 followed by immunoblotting with anti-acetylated-lysine antibody. (B) Caveolar expression were measured by FACS. (C) CTLA4 surface expression and percentages of Tregs for 3 experiments examined. (D-F) HC-derived CD4^+^ T cells were cultured under Treg-polarizing condition. Deacetylase activator Nicotinamide riboside (NR) 1 mM was added on day 3 for 24 h. (D) CPT2 acetylation was determined by a Co-immunoprecipitation assay with anti-CPT2 followed by immunoblotting with anti-acetylated-lysine antibody. (E) Caveolar expression were measured by FACS. (F) CTLA4 surface expression and percentages of Tregs for 3 experiments examined.

**
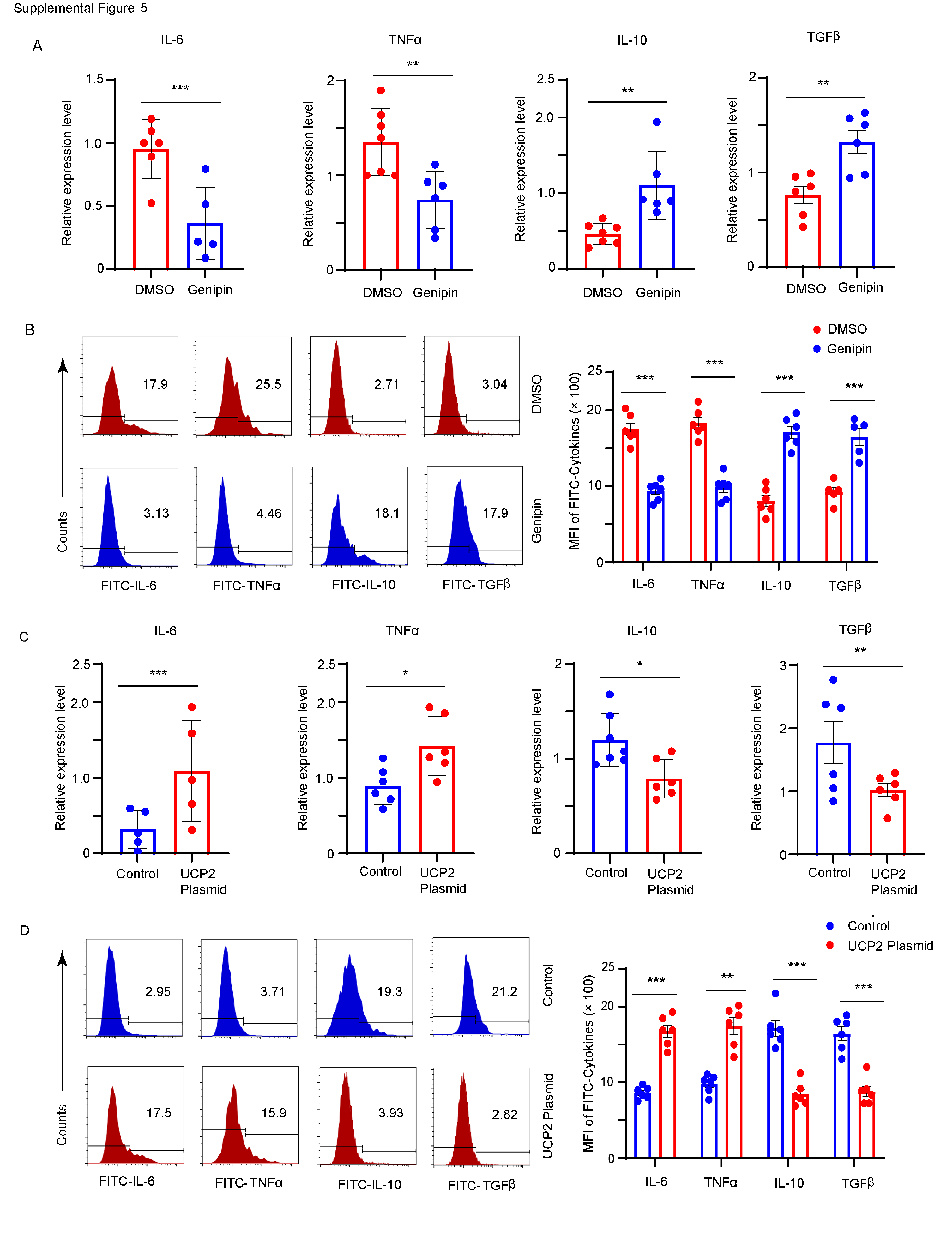
**

**Supplemental Figure S5.** **UCP2 controls the arthritogenic effect of T cells.** Pairs of NSG mice were engrafted with synovial tissue from RA patients, CD45RO^−^ PBMCs from HCs were transferred to the chimeric mice. (A-B) The mice were divided into two groups, vehicle (DMSO) (n=10) and Genipie (n=10) groups. (A) The intensity of synovial inflammation was compared by qPCR to assess *IL-6*, *TNFα*, *IL-10* and *TGFβ* gene expression in each group. (B) IL-6, TNFα, IL-10 and TGFβ Protein expression was analyzed by FACS. (C-D) CD45RO^−^ PBMCs from RA were transfected with either control (n=10) or mCherry-UCP2 plasmids (n=10) and adoptively transferred to the chimeric mice. (C) *IL-6, TNFα* *IL-10* and *TGFβ* gene expression was compared by qPCR. (D) IL-6, TNFα, IL-10 and TGFβ Protein expression was analyzed by FACS. All data were presented as the mean ± SEM. *p < 0.05; **p < 0.01; ***p < 0.001.

**
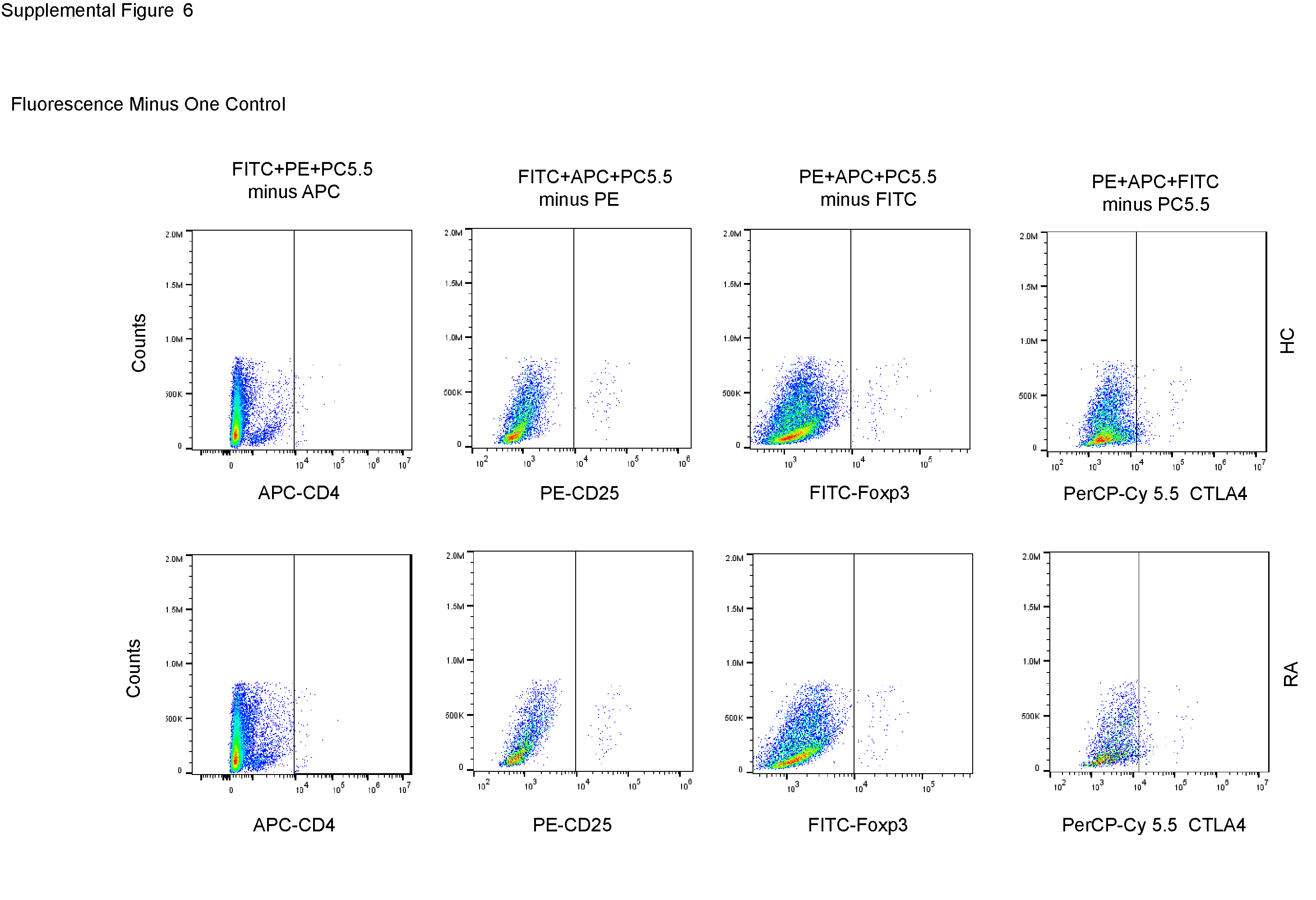
**

**Supplemental Figure S6. Fluorescence Minus One (FMO) control for flow cytometer.** Representative flow cytometry plots of negative controls and FMO controls for each channel, including APC, PE, FITC, and PerCP-Cy5.5, were shown. The experiments were repeated for three times for each plot.

**Supplemental Table S1. Primer sequences for qPCR**

| Target gene | Sense primer (5’-3’) | Antisense primer (5’-3’) |
| --- | --- | --- |
| CAVEOLIN1  CLATHRIN  CPT1A  CPT1B  CPT2  CTLA4  FOXP3  IL6  IL10  PFKFB3  TGFβ  TNFα  TNFSF11  UCP1  UCP2  UCP3  GAPDH | GCGACCCTAAACACCTCAAC  GCACTGAAAGCTGGGAAAACT  TTCAGTTCACGGTCACTCCG  CCTGGTGCTCAAGTCATGGT  CATACAAGCTACATTTCGGGACC  CATGATGGGGAATGAGTTGACC  CAGCCATGATCAGCCTCACA  GGTACATCCTCGACGGCATCT  CCGAGATGCCTTCAGCAGAG  TTGGCGTCCCCACAAAAGT  CCCAGCATCTGCAAAGCTC  TCTTCTCGAACCCCGAGTGA  CCCATAAAGTGAGTCTGTCC  AGGATCGGCCTCTACGACAC  GGAGGTGGTCGGAGATACCAA  AAGGTCCGATTTCAGGCCAG  GGTGGTCTCCTCTGACTTCAACA | ATGCCGTCAAAACTGTGTGTC  CTGCAAGGCTAGAATGGCGA  TGACCACGTTCTTCGTCTGG  CGGTCCAGTTTACGGCGATA  AGCCCGGAGTGTCTTCAGAA  TCAGTCCTTGGATAGTGAGGTTC  CACTGGGATTTGGGAAGGTG  GTGCCTCTTTGCTGCTTTCAC  GGTCTTGGTTCTCAGCTTGG  AGTTGTAGGAGCTGTACTGCTT  GTCAATGTACAGCTGCCGCA  CCTCTGATGGCACCACCAG  CAATACTTGGTGCTTCCTCC  GCCCAATGAATACTGCCACTC  ACAATGGCATTACGAGCAACAT  GCGATGGTTCTGTAGGCGTC  GTTGCTGTAGCCAAATTCGTTGT |

**Supplemental Table S2. Autoantibody panel for Treg suppression assays**

| **Autoantibody** | **HC** | **RA** | **UCP2 inhibitor** | |
| --- | --- | --- | --- | --- |
| Anti-CCP(IU/mL) | 8.72±1.1 | 40.68±5.1 | | 9.63±0.9 |
| RF (IU/mL) | 10.74±2.5 | 56.2±7.5 | | 9.51±1.4 |
| CRP (mg/L) | 4.78±0.7 | 29.2±0.6 | | 3.29±0.8 |
| ASO (IU/mL) | 71.7±8.2 | 220.6±15.4 | | 60.6±7.1 |
| anti-β2GPI IgG (CU) | <6.4 | 11.9±2.2 | | <6.4 |
| anti-β2GPI IgM (CU) | 1.3±0.2 | 6.7±1.3 | | <1.1 |

Anti-CCP, Anti-Cyclic Citrullinated Peptide Antibody; RF, Rheumatoid Factor; CRP, C-Reactive Protein; ASO, Antistreptolysin O; Anti-β2GPI IgG, Anti-Beta-2-Glycoprotein I Antibody IgG; Anti-β2GPI IgM, Anti-Beta-2-Glycoprotein I Antibody IgM.

**Supplemental Table S3. Reagents**

| REAGENTS | | Source | | IDENTIFIER |
| --- | --- | --- | --- | --- |
| Antibodies | | | | |
| Anti-human CD4 APC | | Biolegend | Cat # 300514; RRID:AB_314082 | |
| Anti-human CD45RA PE | | Biolegend | Cat # 304108; RRID:AB_314412 | |
| Anti-humanCD45RA FITC | | Biolegend | Cat # 304106; RRID:AB_314410 | |
| Anti-human CD25 APC | | Biolegend | Cat # 302610; RRID:AB_314280 | |
| Anti-human CD25 PE | | Biolegend | Cat # 302606; RRID:AB_314276 | |
| Anti-human CTLA4 APC | | Biolegend | Cat # 369612; RRID:AB_2632873 | |
| Anti-humanCTLA4-PerCP-Cy5.5 | | Biolegend | Cat # 369608; RRID:AB_2629673 | |
| Anti-human CTLA4 PE | | Biolegend | Cat # 369604; RRID:AB_2566797 | |
| Anti-human FOXP3 FITC | | Biolegend | Cat # 320106; RRID:AB_439752 | |
| Anti-human CD3 | | Abcam | ab21703; RRID:AB_446487 | |
| Anti-human UCP2 antibody | | Santa Cruz | sc-390189; RRID:AB_2721285 | |
| Anti-human CPT1A antibody | | Affinity | DF12004; RRID:AB_2844809 | |
| Anti-human CPT1 antibody | | Santa Cruz | sc-393070; | |
| Anti-human CPT2 antibody | | Santa Cruz | sc-377294; | |
| Anti-human CPT2 antibody | | Affinity | DF 7089 | |
| Anti-human RANKL antibody | | Santa Cruz | sc-59982; RRID:AB_793358 | |
| Anti-human Clathrin heavy chain | | Santa Cruz | sc-12734; RRID:AB_627263 | |
| Anti-human Clathrin light chain | | Santa Cruz | sc-376414; RRID:AB_11149726 | |
| Anti-human caveolin-1 antibody | | Santa Cruz | sc-70516; RRID:AB_1120056 | |
| Anti-human acetylated-lysine | | Santa Cruz | sc-32268; RRID:AB_627898 | |
| Anti-human actin antibody | | Santa Cruz | sc-8432; RRID:AB_626630 | |
| Anti-human CTLA4 | | Santa Cruz | sc-18829; RRID:AB_626919 | |
| [Dynabeads Human T-Activator CD3/CD28](https://www.baidu.com/link?url=Gk9fIMUlaZllhK8TV3QHQm3_1XtCT9XbnSIXQ-HsK4ylldcboTgwJw03ISS1oOxXA1AepOzDmMgB5yHtTyTwyq&wd=&eqid=c94359d60010b19700000006660b77df) | | Invitrogen, | 11132D; RRID:AB_2943359 | |
| Alexa Fluor 594 AffiniPure Donkey Anti-Rabbit IgG (H+L) | | Jackson | 711-585-152; RRID:AB_2340621 | |
| Alexa Fluor 647 AffiniPure Donkey Anti-Rabbit IgG (H+L) | | Jackson | 711-607-003; RRID:AB_2340626 | |
| Alexa Flour 488 AffiniPure Donkey anti-Mouse | | Jackson | 715-545-150; RRID:AB_2340846 | |
| Alexa Fluor 594 AffiniPure Donkey Anti-Mouse IgG (H+L) | | Jackson | 715-585-151; RRID:AB_2340855 | |
| Alexa Fluor 647 AffiniPure Donkey Anti-Mouse IgG (H+L) | | Jackson | 715-607-003; RRID:AB_2340867 | |
| Peroxidase-AffiniPure Goat Anti-rabbit IgG（H+L） | | Jackson | | 111-035-003; RRID:AB_2313567 |
| Peroxidase-AffiniPure Goat Anti-Mouse IgG（H+L） | | Jackson | | 115-035-003; RRID:AB_10015289 |
| Microbeads | | | | |
| CD4 microbeads | Miltenyi Biotec Inc. | | | 130-097-048; |
| CD45RO microbead | Miltenyi Biotec Inc. | | | 130-046-001 |
| Whole blood Treg isolation kit | Miltenyi Biotec Inc. | | | 130-109-557 |
| Chemical, reagents, recombination protein | | | | |
| DAPI | Beyotime | | | C1002 |
| Lymphocyte Separation Medium | Mediatech, Inc., | | | 25-072-CV; |
| IL-2 | Peprotech | | | Cat # 200-02 |
| TGF-β1 | Peprotech | | | Cat # 100-21 |
| CFSE Cell Division Tracker Kit | Biolegend | | | Cat # 423801 |
| RevertAid First strand cDNA Kit | Thermo Scientific | | | K1622 |
| BeyoECL Plus | Beyotime | | | P0018S |
| 3PO | MedChemExpress | | | HY-19824 |
| Genipin | MedChemExpress | | | HY-17389 |
| Etomoxir | MedChemExpress | | | HY-50202 |
| 2,2,6,6-Tetramethylpiperidinooxy (TEMPO) | MedChemExpress | | | HY-100561 |
| N-acetyl-L-cysteine (NAC) | Sigma-Aldrich | | | A9165 |
| H2DCFDA | MedChemExpress | | | HY-D0940 |
| Mitochondrial membrane potential assay kit with JC-1 | Abcam | | | Ab113850 |
